# Vitamin D Metabolism-Related Gene Haplotypes and Their Association with Metabolic Disturbances Among African-American Urban Adults

**DOI:** 10.1038/s41598-018-26230-w

**Published:** 2018-05-23

**Authors:** May A. Beydoun, Sharmin Hossain, Salman M. Tajuddin, Jose A. Canas, Marie Kuczmarski, Hind A. Beydoun, Michele K. Evans, Alan B. Zonderman

**Affiliations:** 1 Laboratory of Epidemiology and Population Sciences, National Institute on Aging, NIA/NIH/IRP, Baltimore, MD United States; 2 Pediatric Endocrinology, Nemours Children’s Clinic, Jacksonville, FL United States; 3 Department of Behavioral Health and Nutrition, University of Delaware, Newark, DE United States; 4 Department of Medicine, Johns Hopkins School of Medicine, Baltimore, MD United States

## Abstract

Epidemiological studies have confirmed associations of the vitamin D receptor (*VDR*) and vitamin D-related gene polymorphisms with adiposity and other metabolic disturbances. Those associations may be sex-specific. We evaluated the cross-sectional and longitudinal relationships between metabolic disturbances and haplotypes constructed from single nucleotide polymorphisms of *VDR* (BsmI:G/A: rs1544410; ApaI:A/C: rs7975232; and TaqI:G/A: rs731236) and *MEGALIN* (rs3755166:G/A; rs2075252:C/T and rs2228171:C/T) genes, in a sample of African-American adults. From 1,024 African Americans participating in the Healthy Aging in Neighborhoods of Diversity across the Life Span (HANDLS, 2004–2013, Baltimore, MD), our analyses included 539 participants with complete genetic, baseline covariate and metabolic outcome data (at baseline and follow-up). Mean ± SD period of follow-up was 4.64 ± 0.93 y. Multivariable-adjusted Cox proportional hazards and logistic regression models were conducted. Among key findings, in men, incident hypertension was inversely related to *MEGALIN*_1_ (GCC), [HR = 0.45, 95% CI: 0.23–0.90, p = 0.024]. Overall, there was a direct, linear dose-response association between *VDR*_2_ (AAG: BAt) and MetS at baseline [OR = 1.60, 95% CI: 1.11–2.31, p = 0.012], while among men, *VDR*_3_ (GAA: bAT) was inversely related to baseline MetS [OR = 0.40, 95% CI: 0.19–0.81, p = 0.011]. In conclusion, *VDR* and *MEGALIN* gene variations can affect prevalent MetS and the incidence rate of hypertension, respectively, among African-American urban adults.

## Introduction

The metabolic syndrome (MetS), is a condition that often clusters together central obesity, elevated blood pressure, lower HDL cholesterol, hypertriglyceridemia and hyperglycemia^1^. MetS increases type 2 diabetes risk and that of cardiovascular disease by 5-folds and 1.7-folds, respectively^2,3^. MetS is heritable and polygenic^4^. Genetics contributes to 16%-85% of Body Mass Index (BMI) variability^5^ and 37%-81% in that of waist circumference (WC) (e.g^6^.). MetS is an important public health threat triggering higher disability, health care costs and mortality from all causes^7–9^.

Moreover, obesity may be directly involved in the etiology of vitamin D deficiency, with prior evidence of an inverse relationship between serum 25-hydroxyvitamin D [25(OH)D] concentration and various measures of adiposity^10^. Conversely, vitamin D3 may influence obesity risk by modulating intracellular calcium homeostasis, due to the fact that higher intracellular calcium triggers lipogenesis and suppresses lipolysis^11^. Many organs express vitamin D receptor (VDR), a component the super-family termed “nuclear hormone receptor”. The complex made of VDR and 1,25(OH)_2_D_3_ modulates transcription of vitamin D responsive genes^12^ and influences adipocyte differentiation^13^. The effect of *VDR* gene polymorphism can potentially be sex-specific as shown in at least one previous study with adiposity phenotypes^14^.

Epidemiological studies have confirmed associations of *VDR* polymorphisms with adiposity and other metabolic disturbances^6,14–23^. However, studies specifically examining adiposity outcomes either had small sample sizes (<400), (e.g^15,16,24^.) or were restricted to one sex, (e.g.^6,16^.) but more importantly were all cross-sectional or case-control by design and none to date have examined these associations among African-American adults.

MEGALIN (aka low-density lipoprotein receptor-related protein-2 [LRP-2]), is the endocytic vitamin D-binding protein receptor which allows vitamin D entry into cells and whose expression is directly regulated by both vitamin D^25^) and vitamin A^26^. MEGALIN may influence obesity by mediating the transport of leptin through the blood-brain barrier and modulating its signaling of both leptin and thyroid hormones^27^ Collectively, leptin and thyroid hormones affect adiposity through energy metabolism regulation^28^. MEGALIN acting also as the receptor for sex-hormone binding globulin (*SHBG*), is involved in interactions between estrogen, vitamin D and intracellular calcium within adipocytes, leading to a potentially sex-specific effect of *MEGALIN* polymorphisms on various phenotypes of obesity, as indicated by findings from previous studies^14,29^

In this study conducted, we hypothesize that selected *VDR* and *MEGALIN* gene polymorphisms have sex-specific associations with several key metabolic disturbances in a longitudinal study of African-American urban adults.

## Subjects and Methods

### Database

The Healthy Aging in Neighborhoods of Diversity across the Life Span (HANDLS) study is a prospective cohort study, initiated in 2004. It recruited an area probability sample of African Americans and whites residing in 13 neighborhoods of Baltimore, Maryland and aged 30–64 years at baseline. In the baseline visit (visit 1: 2004–2009), screening, followed by recruitment and household interviews were completed during phase 1, while phase 2 consisted of in-depth examinations in a mobile Medical Research Vehicles (MRV)^30^. Phase 1 of visit 1 included a general household questionnaire and 1 24 hr dietary recall, while phase 2 of that visit collected more in-depth psychosocial data, anthropometric, physiologic and body composition measurements, a fasting blood draw, as well as a second 24 hr dietary recall. The first follow-up visit (visit 2), initiated in 2009, collected similar data as in phase 2 of visit 1 through 2013, with few variations and followed a similar protocol. In both visits, participants provided informed consent form after reviewing a protocol booklet and a video that explained study procedures including future contact efforts. The National Institute on Environmental Health Sciences Institutional Review Board of the National Institutes of Health approved the HANDLS protocol and all methods were performed in accordance with the relevant guidelines and regulations. Participants are remunerated. In this study, we analyzed longitudinal HANDLS data from initial and first follow-up examinations among a sample of African-Americans participating in the HANDLS study, who had complete genetic data. Time elapsed between examination visit 1 (Wave 1:2004–2009) and visit 2 (also known as Wave 3:2009–2013^31^), ranged between <1 y and ~8 y, with a mean of 4.64 ± 0.93 y.

### Study subjects

**Figure 1:**
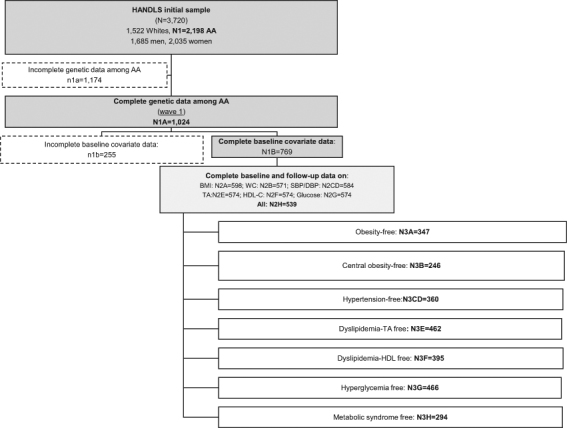
Participant Flow Chart.

Of the 3,720 baseline participants (mean ± SD age(y) of 48.3 ± 9.4, 45.3% men, and 59.1% African-American), data on genetic polymorphisms were complete for 1,024 participants self-reporting to be African American. However, missing data on covariates reduced our sample to n = 769, and further exclusions resulted in a sample size range of 574 to 598, with 539 participants having complete data on relevant baseline and follow-up outcome measurements (cross-sectional part of the analysis). In the longitudinal part of our analyses, participants who were initially free from metabolic disturbances were selected for each outcome. Their sample sizes ranged from n = 246 (central obesity-free) to n = 466 (hyperglycemia-free) and those who were MetS-free consisted of n = 294 baseline participants (Fig. 1).

### Anthropometric measures and metabolic outcome variables

BMI, measured as weight/height^2^, kg/m^2^ was computed for each participant based on measured weight and height. Furthermore, WC (in cm.) was measured using a tape that was wrapped around the waist near the navel, starting from the hip bone. Systolic and diastolic blood pressures (SBP and DBP) were measured by averaging 1 right and 1 left sitting non-invasive assessments using brachial artery auscultation using a stethoscope, an aneroid manometer, and an inflatable cuff. After an overnight fast (8–12 hours), a blood draw was taken from an antecubital vein. From this blood draw, fasting glucose (FG), triacylglycerols (TAG), total cholesterol, and HDL-C were assessed using a spectrophotometer (Olympus 5400; Quest Diagnostics).

### Classification of health outcomes

General obesity was defined as BMI ≥30 kg/m^2^, while central obesity (aka abdominal obesity) was based on WC ≥102 cm or 40 inches in men and ≥88 cm or 35 inches in women^32^. Participants who screened positive on at least 3 of 5 conditions ((1) central obesity (see above); (2) blood pressure ≥130/85 mmHg; (3) dyslipidemia: TAG ≥1.695 mmol/L (150 mg/dl); (4) dyslipidemia: HDL-C < 40 mg/dL in men or <50 mg/dL in women; (5) fasting plasma glucose ≥6.1 mmol/L (110 mg/dl)^33^.) were classified as MetS-positive^1^ We examined binary prevalent (V1 and V2) and incident outcomes, namely obesity, central obesity, MetS and its remaining individual components (i.e. hypertension, dyslipidemia-TA, dyslipidemia-HDL and hyperglycemia).

#### Vitamin D receptor and MEGALIN (LRP2) SNP and SNPHAP

Study participants were genotyped to 907,763 single nucleotide polymorphisms (SNPs) using the Illumina 1 M and 1M-Duo genotyping arrays. Details regarding genotype quality control criteria are provided in Supplemental Methods 1.

For the present study, in the main analysis, we selected *VDR* and *MEGALIN* SNPs based on previously published validation studies of adiposity or various health outcomes that were linked to adiposity^6,15–18^ and a replication study of similar outcomes among European ancestry participants from the Baltimore Longitudinal Study of Aging (BLSA)^14^. Three *VDR* SNPs:; rs1544410 (BsmI: G/A); rs7975232 (ApAI: A/C) and rs731236 (TaqI: G/A), and three *MEGALIN* SNPs (rs3755166: G/A; rs2075252: C/T; rs2228171: C/T) were chosen for haplotype analysis. The final selected SNPs and their frequencies were published elsewhere^34^.

*VDR* and *MEGALIN* SNPs haplotypes (SNPHAP) were considered main predictors in our analysis. For *VDR* gene, the BsmI, ApaI and TaqI SNP were combined together to construct SNPHAP, as was done in a previous study^34^, and their haplotype frequencies in the population were comparable to at least one other study conducted among Whites^35^. Four SNPHAP were detected in our sample with the SNP combinations being either one of the three: *VDR*_1_: GCA [baT], *VDR*_2_: AAG [BAt], *VDR*_3_: GAA [bAT] and *VDR*_4_: AAA [BAT] for one or two alleles. Participants were coded as 0 = having no *VDR*_x_ haplotype; 1 = having one allele carrying the *VDR*_*x*_ haplotype; 2 = having two alleles with the *VDR*_x_ haplotype. This approach was similarly applied to the three *MEGALIN* SNP and eight haplotypes were found. However, only two haplotypes were extracted in the present analyses, given that their frequency was greater than 10%. The most common SNPHAP are comparable to our previous study^34^. Detailed descriptions the SNPHAP are found in Table 1. Furthermore, all available SNPs in and around the *VDR* and *MEGALIN* genes were also selected for a supplemental analysis, after passing through eligibility criteria related to reliability of imputation and minor allele frequency. Details on filtering of SNPs is further discussed in Supplemental Method 1–2. Outcomes of interest were MetS (incident, visits 1 and 2).

**Table 1:** Findings from haplotype analysis: definitions and distributions of SNPHAP for the selected *VDR* and *LRP2* (*MEGALIN*) SNPs, *n* = 1,024^1^.

	SNP Haplotypes (SNPHAP)	
	Definitions	Distributions, %
*VDR*	[BsMI/ApaI/TaqI]	
Overall	*VDR*_1_: GCA [baT]	36.5
	*VDR*_2_: AAG [BAt]	19.1
	*VDR*_3_: GAA [bAT]	25.2
	*VDR*_4_: AAA [BAT]	10.1
Allelic copies		
*VDR*_1_	*VDR*_1_: GCA	
0		68.0
1		18.5
2		13.6
*VDR*_2_	*VDR*_2_: AAG	
0		78.5
1		17.3
2		4.2
*VDR*_3_	*VDR*_3_: GAA	
0		27.3
1		65.8
2		6.8
*VDR*_4_	*VDR*_4_: AAA	
0		90.2
1		8.8
2		1.0
*MEGALIN*	[rs3755166/rs2075252/rs2228171]	
Overall		
	*MEGALIN*_1_:GCC	53.3
	*MEGALIN*_2_:ACC	24.3
Allelic copies		
*MEGALIN*_1_	*MEGALIN*_1_:GCC	
0		10.1
1		63.5
2		26.5
*MEGALIN*_2_	*MEGALIN*_2_:ACC	
0		64.9
1		30.8
2		4.3

*Abbreviations*: SNP = Single Nucleotide Polymorphism; SNPHAP = Single Nucleotide Polymorphism Haplotype; *VDR* = Vitamin D receptor gene.

^1^SNPHAP were defined based on three *VDR* SNP combinations: BsmI, ApaI and TaqI and were expressed as dosage (0 = none, 1 = one copy, 2 = 2 copies) in the main analysis. SNPHAP were defined based on all three *MEGALIN* SNP combinations rs3755166/rs2075252/rs2228171 and were expressed as dosage (0 = none, 1 = one copy, 2 = 2 copies) in the main analysis.

### Covariates

Our analyses included the following covariates: baseline age, sex, poverty status, education, smoking, drug use and self-rated health, among fixed or baseline variables. The Healthy Eating Index (HEI-2010) total score, computed using two 24-hr recalls administered at the initial visit, reflected overall dietary quality (see http://appliedresearch.cancer.gov/tools/hei/tools.html) or http://handls.nih.gov/06Coll-dataDoc.htm) and was included in our analyses. Similarly, total energy intake (kcal/d) was included in our models as a potential confounder based on the average of initial 2 24-hr dietary recalls. Finally, 10 principal components were included in order to control for any residual effects of population structure (Supplemental method 1). Covariates were selected based on their known association with the metabolic outcomes of interest. Due to the limited sample size, a sensitivity analysis was conducted for parts of the analysis adjusting only for basic socio-demographic factors, namely age, sex, poverty status and education, as well as the inverse mills ratio.

### Statistical analysis

The main part of the analysis was conducted using Stata release 15.0^36^. For each SNP, the Hardy-Weinberg equilibrium assumption was tested using exact test, and pair-wise linkage disequilibrium (LD) was computed and visualized using Haploview version 4.2 package^37^. To describe study participant characteristics and compare them by sex, *t*-test and χ^2^ test were used for continuous and categorical variables, respectively.

Both cross-sectional and longitudinal relationships of *VDR* and *MEGALIN* SNPHAP with binary metabolic outcomes, including obesity, central obesity and the MetS were examined. To test cross-sectional associations, multi-variable logistic regression models were conducted for each outcome, controlling for baseline age, sex, poverty status, education, first-visit current smoking and drug use, self-reported health and the HEI-2010 total score, the 10 principal components to adjust for population structure and the inverse mills ratio. For longitudinal analyses, we defined time-to-event from baseline visit (i.e. delayed entry) until outcome or censoring at second visit and constructed multiple Cox proportional hazards models for incident metabolic outcome, overall and after stratifying by sex. Follow-up time was expressed in years. In addition to examining obesity (BMI and WC-based) and MetS as incident outcomes, other components of the MetS were also evaluated as individual incident metabolic outcomes. Linear trend test for associations between haplotype dosage (0, 1, 2 copies) and metabolic outcomes was performed.

Furthermore, selection bias due to the non-random selection of participants with genetic data from target population, was corrected at least in part using a 2-stage Heckman selection model^38^. At first stage, probit models were constructed to calculate an inverse mills ratio, a function of the predicted selection probability, conditional on key covariates, as previously described^39^. At a second stage, the inverse mills ratio was entered into the multi-variable logistic or Cox PH models, thus adjusting for selection bias. Stratification was done and effect modification was tested (by adding 2-way interaction terms) by sex for all analyses, including supplemental analyses for single SNPs in and around the *VDR* and *MEGALIN* genes. Gender difference in the relationship between *MEGALIN* gene polymorphisms and metabolic outcomes was an a priori hypothesis^40^.

Finally, in all our analyses, type I error was set 0.05 prior to correction for multiple testing. A p-value <0.10 was considered as marginally significant. Correction for multiple testing was conducted using a familywise Bonferroni process in which a family was defined by the metabolic outcome and the gene^41^. Thus, the critical p-value was reduced to 0.05/4 = 0.0125 in the case *VDR* SNPHAP associations, whereas for *MEGALIN* SNPHAPs it was reduced to 0.05/2 = 0.025. Correction for multiple testing followed a similar though less stringent approach, whereby a critical p-value per outcome of interest was reduced to 0.01 for overall analysis and 0.02 for sex-specific analysis. For 2-way interaction terms, particularly for testing effect modification by gender, type I error was kept at 0.05 due to reduced statistical power^42^.

## Results

All examined SNPs exhibited Hardy-Weinberg equilibrium (P > 0.002). Variants within each *VDR* and *MEGALIN* (*LRP2*) gene were deemed in low linkage equilibrium (r^2^ < 0.30). The four selected *VDR* haplotypes had an overall prevalence ranging from 10.1% for *VDR*_4_ to 36.5% for *VDR*_1_. A large proportion of African-Americans (65.8%) had 1 copy of *VDR*_3_; only 1% had 2 copies of *VDR*_4_. Similarly, among the selected *MEGALIN* haplotypes, *MEGALIN*_1_: GCC was the most common (53.3%), with 63.5% having 1 copy and only 4.3% having 2 copies of *MEGALIN*_2_: ACC (Table 1).

Table 2 presents baseline characteristics and time-dependent metabolic outcomes (Fig. 1: n_2h_ = 539). Most notably men had higher prevalence of smoking and drug use compared to women as well as higher energy intake, poorer overall dietary quality (HEI-2010 total score), and higher mean fasting blood glucose. In contrast, women had higher mean BMI, WC, HDL-C compared to men, at both waves. Among incident outcomes, central obesity was markedly higher among women compared to men, with no difference noted for incident obesity or incident MetS. Nevertheless, in the cross-sectional data, obesity and central obesity were both significantly more prevalent among women compared to men at both waves, while MetS prevalence was higher among women only at follow-up.

**Table 2:** Gender differences in baseline characteristics and time-dependent metabolic outcomes among African-Americans with complete genetic, time-dependent metabolic data and baseline covariate data: HANDLS 2004–2009 and 2009–2013^1^.

	All		Men		Women	
	(*n* = 539)		(*n* = 230)		(*n* = 309)	
	Mean, %	SE	Mean, %	SE	Mean, %	SE
**Socio-demographic and health characteristics, V1**						
Age (y)	48.6	0.4	49.0	0.6	48.3	0.5
Men (%)	42.7		__		__	
Above poverty (%)	52.5		54.8		50.8	
Education (%)						
<High School	3.9		4.3		3.6	
High School	61.2		59.6		62.5	
>High School	34.9		36.1		34.0	
Self-rated health (%)						
Poor/fair	21.3		20.0		22.3	
Good	44.3		45.2		43.7	
Very good/excellent	34.3		34.8		34.0	
Current smoker, yes (%)	45.3		51.3^2^		40.8	
Current smoker, missing (%)	5.0		3.9		5.8	
Current illicit drug user, yes (%)	18.6		23.9^2^		14.6	
Current illicit drug user, missing (%)	5.0		3.9		5.8	
**Dietary intake and quality**, **V1**						
Energy intake (kcal/d)	2,033	43	2408^2^	74	1755	45.4
HEI-2010	42.8	0.5	41.7^2^	0.7	43.7	0.6
**Metabolic outcomes, V1, V2**						
BMI (kg/m^2^)						
V1	29.9	0.3	28.0^2^	0.4	31.3	0.5
V2	30.5	0.3	28.3^2^	0.4	32.2	0.5
Waist circumference (cm)						
V1	98.6	0.8	96.7^2^	1.1	100.1	1.1
V2	102.3	0.7	100.1^2^	1.0	103.9	1.1
SBP (mm Hg)						
V1	122.1	0.7	121.2	1.0	122.8	1.1
V2	124.8	0.8	123.0^2^	1.2	126.2	1.1
DBP (mm Hg)						
V1	73.2	0.5	74.0	0.7	72.6	0.6
V2	71.9	0.4	72.7	0.7	71.2	0.5
HDL-C (mg/dL)						
V1	55.0	0.7	51.5^2^	1.1	57.7	1.0
V2	59.1	0.8	54.7^2^	1.2	62.4	1.1
TA (mg/dL)						
V1	107.0	3.3	116.1^2^	6.5	100.2	2.9
V2	110.8	2.6	113.9	4.3	108.5	3.1
Fasting blood glucose (mg/dL)						
V1	104.1	1.7	108.5^2^	3.1	100.8	1.8
V2	103.2	1.5	107.1^2^	2.6	100.3	1.7
**Metabolic disturbance**, **V1, V2, incident**						
Obesity (%, BMI ≥ 30)						
V1	41.2		30.0^2^		49.5	
V2	46.8		33.9^2^		56.3	
Incident	15.0		11.5		18.5	
Central obesity (%)^3^						
V1	57.7		36.5^2^		73.5	
V2	66.4		43.0^2^		83.8	
Incident	31.7		21.7^2^		49.4	
MetS (%)^4,5^						
V1	24.9		22.2		26.9	
V2	22.8		18.7^2^		25.9	
Incident	12.2		9.8		14.3	

^1^HANDLS, Healthy Aging in Neighborhoods of Diversity across the Life Span; SBP, systolic blood pressure; DBP, diastolic blood pressure; TA, triacylglycerols; MetS, metabolic syndrome, V1 = Visit 1, V2 = Visit 2.

^2^P < 0.05 for testing the null hypothesis that means or proportions are the same between men and women.

^3^Defined as waist circumference > 102 cm for men and > 88 cm for women.

^4^Defined based on NCEP ATP III described in Methods.

^5^Three or more metabolic disturbances as listed above represent MetS. Metabolic disturbances may range between 0 and 5.

Table 3 shows associations of *VDR* and *MEGALIN* haplotypes with incident metabolic disturbances (obesity, MetS, and individual MetS components), stratifying by sex. Among all key results, only survived correction for multiple testing. In fact, among men, incident hypertension was inversely related to the *MEGALIN*_1_ haplotype (HR = 0.45, 95% CI:0.23–0.90, p = 0.024). Though not surviving correction for multiple testing, this haplotype was also inversely related to incident MetS among men. Similarly, incident hyperglycemia was linked to *VDR*_1_ haplotype in men. Among women, *VDR*_3_ was inversely related to incident central obesity, and *VDR*_2_ was directly associated with incident hypertension. *MEGALIN*_2_ was consistently inversely related to incident hypertension and incident hyperglycemia among women. The latter association differed significantly between sexes.

**Table 3:** *VDR* and *MEGALIN* SNP haplotype (SNPHAP) associations with incident metabolic disturbances: Cox proportional hazards models, (*n* = 246–466); HANDLS study.

	Incident metabolic disturbance									
	All			Men				Women		
	HR	95%CI	*P*	HR		95%CI	*P*	HR	95%CI	*P*
**Obesity: Models A-F**	**(N = 347)**			**(N = 174)**				**(N = 173)**		
*VDR*_1_: GCA (0,1,2)	1.30	(0.87;1.93)	0.20	1.19		(0.69;2.07)	0.54	1.19	(0.69;2.07)	0.54
*VDR*_2_: AAG (0,1,2)	0.89	(0.48;1.63)	0.71	1.02		(0.35;2.94)	0.97	0.97	(0.35;2.64)	0.95
*VDR*_3_: GAA (0,1,2)	1.21	(0.66;2.20)	0.55	1.21		(0.34;4.31)	0.76	1.49	(0.63;3.48)	0.36
*VDR*_4_: AAA(0,1,2)	0.74	(0.27;2.03)	0.57	0.83		(0.14;4.91)	0.84	0.51	(0.11;2.45)	0.40
*MEGALIN*_1_: GCC (0,1,2)	0.94	(0.55;1.61)	0.83	0.50		(0.17;1.48)	0.21	1.59	(0.74;3.42)	0.23
*MEGALIN*_2_: ACC (0,1,2)	1.23	(0.72;2.10)	0.45	1.51		(0.58;3.96)	0.40	0.63	(0.36;1.50)	0.30
**Central obesity: Models A-F**	**(N** = **246)**			**(N = 157)**				**(N = 89)**		
*VDR*_1_: GCA (0,1,2)	1.19	(0.82;1.74)	0.37	1.00		(0.49;2.01)	1.00	**1** ***.63***	***(0.96;*** **2** ***.76)***	***0.068***
*VDR*_2_: AAG (0,1,2)	1.02	(0.62;1.70)	0.93	0.83		(0.41;1.71)	0.62	1.38	(0.68;3.63)	0.29
*VDR*_3_: GAA (0,1,2)	0.81	(0.49;1.35)	0.42	1.60		(0.60;4.24)	0.35	**0.47**	**(0.23;0.95)**	**0.036**
*VDR*_4_: AAA(0,1,2)	***1.57***	***(0.97;2.52)***	***0.064***	1.91		(0.78;4.66)	0.16	1.34	(0.58;3.09)	0.49
*MEGALIN*_1_: GCC (0,1,2)	0.86	(0.56;1.31)	0.49	0.57		(0.27;1.20)	0.14	0.92	(0.48;1.73)	0.80
*MEGALIN*_2_: ACC (0,1,2)	1.27	(0.81;1.98)	0.30	**2.16**		**(1.04;4.32)**	**0.040**	0.90	(0.45;1.82)	0.78
**Hypertension: Models A-F**	**(N = 360)**			**(N = 159)**				**(N = 201)**		
*VDR*_1_: GCA (0,1,2)	1.08	(0.77;1.51)	0.67	0.88		(0.49;1.57)	0.66	1.45	(0.91;2.31)	0.12
*VDR*_2_: AAG (0,1,2)	1.30	(0.86;1.97)	0.21	1.03		(0.50;2.13)	0.93	**1.98**	**(1.09;3.62)**	**0.026**
*VDR*_3_: GAA (0,1,2)	1.00	(0.62;1.59)	0.99	1.45		(0.61;3.43)	0.40	0.59	(0.30;1.14)	0.12
*VDR*_4_: AAA(0,1,2)	0.86	(0.46;1.62)	0.65	1.37		(0.54;3.49)	0.50	0.81	(0.30;2.15)	0.67
*MEGALIN*_1_: GCC (0,1,2)	0.77	(0.52;1.12)	0.17	**0.45**		**(0.23;0.90)**	**0.024**	1.08	(0.59;1.96)	0.81
*MEGALIN*_2_: ACC (0,1,2)	0.84	(0.57;1.22)	0.36	1.28		(0.70;2.36)	0.43	**0.55**	**(0.31;0.97)**	**0.039**
**Dyslipidemia-TA: Models A-F**	**(N** = **462)**			**(N** = **183)**				**(N** = **279)**		
*VDR*_1_: GCA (0,1,2)	***1.4*** **3**	***(0.95;2*** *.* **1** ***6)***	***0.085***	1.37		(0.86;2.86)	0.14	1.42	(0.71;2.83)	0.33
*VDR*_2_: AAG (0,1,2)	1.08	(0.67;1.76)	0.75	0.90		(0.37;2.23)	0.82	1.30	(0.62;2.70)	0.49
*VDR*_3_: GAA (0,1,2)	0.82	(0.47;1.42)	0.47	0.58		(0.24;1.42)	0.23	0.87	(0.39;1.90)	0.72
*VDR*_4_: AAA(0,1,2)	0.59	(0.23;1.56)	0.29	0.96		(0.24;3.79)	0.96	0.43	(0.09;2.02)	0.28
*MEGALIN*_1_: GCC (0,1,2)	0.95	(0.59;1.53)	0.84	1.03		(0.46;2.29)	0.94	1.23	(0.58;2.58)	0.59
*MEGALIN*_2_: ACC (0,1,2)	0.90	(0.55;1.47)	0.67	0.80		(0.31;2.06)	0.65	0.66	(0.83;1.32)	0.24
**Dyslipidemia-HDL: Models A-F**	**(N** = **395)**			**(N** = **184)**				**(N** = **211)**		
*VDR*_1_: GCA (0,1,2)	1.23	(0.68;2.19)	0.49	1.36		(0.48;3.81)	0.36	1.05	(0.30;3.65)	0.94
*VDR*_2_: AAG (0,1,2)	0.37	(0.11;1.26)	0.11	0.66		(0.15;2.82)	0.57	0.17	(0.01;2.51)	0.20
*VDR*_3_: GAA (0,1,2)	0.89	(0.40;2.01)	0.79	0.76		(0.18;3.27)	0.71	0.72	(0.16;3.16)	0.66
*VDR*_4_: AAA(0,1,2)	1.24	(0.37;2.64)	0.59	0.50		(0.07;3.76)	0.50	2.47	(0.78;7.83)	0.12
*MEGALIN*_1_: GCC (0,1,2)	0.70	(0.36;1.38)	0.30	0.81		(0.26;2.49)	0.71	0.73	(0.24;2.24)	0.58
*MEGALIN*_2_: ACC (0,1,2)	1.67	(0.90;3.10)	0.11	2.34		(0.80;6.85)	0.12	1.30	(0.46;3.62)	0.63
**Hyperglycemia: Models A-F**	**(N** = **466)**			**(N = 187)**				**(N = 279)**		
*VDR*_1_: GCA (0,1,2)	***1.51***	(***0.99;*2*.29***)	***0.054***	**2.26**		**(1.11;4.62)**	**0.025**	1.08	(0.57;2.04)	0.80
*VDR*_2_: AAG (0,1,2)	0.54	(0.25;1.14)	0.11	0.97		(0.34;2.77)	0.96	0.49	(0.16;1.45)	0.20
*VDR*_3_: GAA (0,1,2)	0.88	(0.47;1.64)	0.69	0.67		(0.22;2.05)	0.49	0.89	(0.36;2.23)	0.81
*VDR*_4_: AAA (0,1,2)	0.81	(0.35;1.88)	0.63	0.39		(0.04;3.69)	0.41	1.92	(0.65;5.68)	0.24
*MEGALIN*_1_: GCC (0,1,2)	0.89	(0.51;1.53)	0.67	***0.40***		***(0.14;1.14)***	***0.087***	1.09	(0.52;2.28)	0.81
*MEGALIN*_2_: ACC (0,1,2)	0.74	(0.41;1.33)	0.32	1.31^3^		(0.52;3.32)	0.57	**0.39**	**(0.16;0.97)**	**0.043**
**Metabolic syndrome: Models A-F**	**(N** = **294)**			**(N** = **133)**				**(N** = **161)**		
*VDR*_1_: GCA (0,1,2)	0.86	(0.44;1.66)	0.64	1.16		(0.37;3.65)	0.79	0.88	(0.33;2.39)	0.81
*VDR*_2_: AAG (0,1,2)	0.89	(0.36;2.01)	0.78	0.92		(0.13;6.58)	0.93	1.02	(0.32;3.22)	0.97
*VDR*_3_: GAA (0,1,2)	1.99	(0.87;4.53)	0.10	1.24		(0.19;8.02)	0.82	2.34	(0.66;8.30)	0.19
*VDR*_4_: AAA(0,1,2)	***0.18***	***(0.02;1.27)***	***0.085***	2.42		(0.27;21.90)	0.43	__		
*MEGALIN*_1_: GCC (0,1,2)	0.88	(0.46;1.70)	0.71	**0.08**		**(0.01;0.88)**	**0.039**	0.91	(0.30;2.72)	0.86
*MEGALIN*_2_: ACC (0,1,2)	0.95	(0.50;1.81)	0.88	0.94		(0.19;4.69)	0.94	1.02	(0.37;2.81)	0.97

*Abbreviations*: BMI = body mass index (calculated as weight in kg/square of height in meters); SNP = Single Nucleotide polymorphism; SNPHAP = SNP haplotype; *VDR* = Vitamin D receptor gene; Note that *VDR*_1_*, VDR*_2_*, VDR*_3_ denote *VDR* SNPHAP, whereas *MEGALIN*_1_, *MEGALIN*_2_ and *MEGALIN*_3_ denote *MEGALIN* SNPHAP.

*Note*: Shaded estimated indicate significance upon correction for multiple testing. Models A-F indicate that each haplotype was entered in a separate regression model to estimate its association with different metabolic outcomes.

^1^See Table 1 for more details on definition the SNP haplotypes. (0,1,2) refers to ordinal coding with “0”, “1” and “2” copies of each haplotype. Three *VDR* SNP were combined to form the haplotypes, namely BsmI, ApaI and TaqI. Only haplotypes 1 and 2 were selected for *MEGALIN* since their overall prevalence was > 10%.

^2^Models were adjusted for age, sex, poverty status, education, current smoking status, current illicit drug use, self-rated health, total energy intake, HEI-2010 total score, 10 principal components for population structure, and the inverse mills ratio.

^3^P < 0.05 for null hypothesis that sex × SNPHAP interaction term = 0 in a model where main effect of sex was added.

Cross-sectional associations between the selected *VDR* and *MEGALIN* haplotypes and the main metabolic disturbances are presented in Tables 4 (baseline outcomes) and 5 (follow-up outcomes). There was a linear dose-response direct association between *VDR*_2_ and prevalent obesity and MetS at baseline, with no significant sex differences, (OR = 1.60, 95% CI:1.11–2.31, p = 0.012). Moreover, *VDR*_3_ was inversely related to prevalent MetS at baseline among men, (OR = 0.40, 95% CI:0.19–0.81, p = 0.011), an association that differed significantly by sex (P < 0.05 for sex × SNPHAP interaction term). Both of these findings survived correction for multiple testing. The associations of *VDR* and *MEGALIN* SNPHAPs with follow-up outcomes did not survive correction for multiple testing. Among those, *VDR*_2_ was positively associated with prevalent obesity, overall and among women, a finding consistent with the baseline outcome. *MEGALIN* haplotypes were not associated with prevalent baseline or follow-up outcomes of obesity, central obesity and MetS. A sensitivity analysis that included only basic socio-demographic factors yielded similar results.

**Table 4:** VDR and MEGALIN SNP haplotype (SNPHAP) associations with prevalent metabolic disturbances (V1): multiple logistic regression models, (*n* = 539); HANDLS study.

	Prevalent metabolic disturbance								
	All (N = 539)			Men (N = 230)			Women (N = 309)		
	OR	95%CI	*P*	OR	95%CI	*P*	OR	95%CI	*P*
**Obesity: Models A-F**									
*VDR*_1_: GCA (0,1,2)	0.88	(0.67;1.15)	0.36	1.18	(0.75;1.86)	0.46	0.76	(0.53;1.10)	0.14
*VDR*_2_: AAG (0,1,2)	**1.44**	**(1.02;2.03)**	**0.038**	1.22	(0.70;2.14)	0.49	***1*** **.50**	***(0.94;*** **2** ***.40)***	***0.089***
*VDR*_3_: GAA (0,1,2)	0.95	(0.67;1.35)	0.78	0.74	(0.40;1.36)	0.34	1.04	(0.66;1.65)	0.84
*VDR*_4_: AAA(0,1,2)	1.21	(0.75;1.95)	0.44	1.21	(0.56;2.63)	0.63	1.30	(0.67;2.50)	0.43
*MEGALIN*_1_: GCC (0,1,2)	1.11	(0.80;1.56)	0.53	0.86	(0.48;1.53)	0.61	1.16	(0.74;1.82)	0.51
*MEGALIN*_2_: ACC (0,1,2)	0.95	(0.70;1.32)	0.78	1.00	(0.58;1.73)	1.00	1.03	(0.67;1.60)	0.90
**Central obesity: Models A-F**									
*VDR*_1_: GCA (0,1,2)	0.88	(0.70;1.16)	0.35	1.31^3^	(0.85;2.03)	0.22	**0.66**	**(0.44;0.98)**	**0.040**
*VDR*_2_: AAG (0,1,2)	1.36	(0.93;1.99)	0.12	1.21	(0.71;2.05)	0.50	***1.76***	***(0.92;*** **3** ***.36)***	***0.083***
*VDR*_3_: GAA (0,1,2)	1.14	(0.78;1.70)	0.50	0.80	(0.42;1.36)	0.36	1.37	(0.79;2.36)	0.26
*VDR*_4_: AAA(0,1,2)	0.88	(0.53;1.48)	0.64	1.06	(0.48;2.34)	0.88	0.80	(0.38;1.69)	0.56
*MEGALIN*_1_: GCC (0,1,2)	1.31	(0.91;1.88)	0.14	1.16	(0.68;2.02)	1.00	1.30	(0.78;2.17)	0.32
*MEGALIN*_2_: ACC (0,1,2)	0.93	(0.66;1.32)	0.69	0.91	(0.53;1.55)	0.73	1.04	(0.63;1.74)	0.87
**Metabolic syndrome: Models A-F**									
*VDR*_1_: GCA (0,1,2)	0.81	(0.59;1.10)	0.18	0.99	(0.60;1.64)	0.96	0.69	(0.44;1.08)	0.10
*VDR*_2_: AAG (0,1,2)	**1.60**	**(1.11;2.31)**	**0.012**	**1.91**	**(1.05;3.48)**	**0.034**	***1.62***	***(0.97;2.70)***	***0.063***
*VDR*_3_: GAA (0,1,2)	0.89	(0.60;1.31)	0.54	**0.40** ^3^	**(0.19;0.81)**	**0.011**	1.23	(0.74;2.07)	0.43
*VDR*_4_: AAA(0,1,2)	0.85	(0.50;1.48)	0.57	1.09	(0.46;1.99)	0.84	0.70	(0.32;1.56)	0.39
*MEGALIN*_1_: GCC (0,1,2)	0.76	(0.52;1.13)	0.16	0.88	(0.45;1.74)	0.72	***0.64***	***(0.38;1.07)***	***0.091***
*MEGALIN*_2_: ACC (0,1,2)	1.13	(0.79;1.63)	0.50	1.17	(0.62;2.19)	0.64	1.21	(0.74;1.98)	0.45

*Abbreviations*: BMI = body mass index (calculated as weight in kg/square of height in meters); SNP = Single Nucleotide polymorphism; SNPHAP = SNP haplotype; V1 = Visit 1; *VDR* = Vitamin D receptor gene; Note that *VDR*_1_*, VDR*_2_*, VDR*_3_ denote *VDR* SNPHAP, whereas *MEGALIN*_1_, *MEGALIN*_2_ and *MEGALIN*_3_ denote *MEGALIN* SNPHAP.

*Note*: Shaded estimated indicate significance upon correction for multiple testing. Models A-F indicate that each haplotype was entered in a separate regression model to estimate its association with different metabolic outcomes.

^1^See Table 1 for more details on definition the SNP haplotypes. (0,1,2) refers to ordinal coding with “0”, “1” and “2” copies of each haplotype. Three *VDR* SNP were combined to form the haplotypes, namely BsmI, ApaI and TaqI. Only haplotypes 1 and 2 were selected for *MEGALIN* since their overall prevalence was >10%.

^2^Models were adjusted for age, sex, poverty status, education, current smoking status, current illicit drug use, self-rated health, total energy intake, HEI-2010 total score, 10 principal components for population structure, and the inverse mills ratio.

^3^P < 0.05 for null hypothesis that sex × SNPHAP interaction term = 0 in a model where main effect of sex was added.

**Table 5:** VDR and MEGALIN SNP haplotype (SNPHAP) associations with prevalent metabolic disturbances (V2): multiple logistic regression models, (*n* = 539); HANDLS study.

	Prevalent metabolic disturbance									
	All (N = 539)				Men (N = 230)			Women (N = 309)		
	OR		95%CI	*P*	OR	95%CI	*P*	OR	95%CI	*P*
**Obesity: Models A-F**										
*VDR*_1_: GCA (0,1,2)	0.89		(0.68;1.16)	0.37	1.05	(0.68;1.60)	0.83	0.78	(0.55;1.11)	0.17
*VDR*_2_: AAG (0,1,2)	**1.43**		**(1.01;2.02)**	**0.044**	1.08	(0.63;1.84)	0.78	**1.79**	**(1.09;2.95)**	**0.023**
*VDR*_3_: GAA (0,1,2)	0.96		(0.69;1.36)	0.85	0.94	(0.53;1.66)	0.82	0.99	(0.63;1.56)	0.96
*VDR*_4_: AAA(0,1,2)	1.17		(0.72;1.88)	0.53	1.32	(0.62;2.81)	0.47	1.19	(0.61;2.29)	0.61
*MEGALIN*_1_: GCC (0,1,2)	1.08		(0.77;1.50)	0.67	0.95	(0.54;1.67)	0.85	1.13	(0.73;1.77)	0.56
*MEGALIN*_2_: ACC (0,1,2)	1.06		(0.76;1.46)	0.74	1.17	(0.68;1.99)	0.57	1.05	(0.68;1.62)	0.82
**Central obesity: Models A-F**										
*VDR*_1_: GCA (0,1,2)		1.03	(0.76;1.40)	0.83	1.10	(0.72;1.69)	0.65	1.07	(0.66;1.73)	0.79
*VDR*_2_: AAG (0,1,2)		1.18	(0.79;1.77)	0.41	0.90	(0.54;1.52)	0.71	1.75	(0.82;3.77)	0.15
*VDR*_3_: GAA (0,1,2)		1.02	(0.69;1.52)	0.92	1.22	(0.69;2.14)	0.49	0.84	(0.46;1.56)	0.59
*VDR*_4_: AAA(0,1,2)		1.30	(0.73;2.32)	0.37	1.55	(0.71;3.38)	0.27	0.99	(0.41;2.41)	0.98
*MEGALIN*_1_: GCC (0,1,2)		1.04	(0.71;1.53)	0.84	0.77	(0.44;1.35)	0.36	1.35	(0.72;2.52)	0.35
*MEGALIN*_2_: ACC (0,1,2)		1.10	(0.76;1.62)	0.61	1.26	(0.74;2.15)	0.40	1.05	(0.57;1.94)	0.87
**Metabolic syndrome: Models A-F**										
*VDR*_1_: GCA (0,1,2)		0.96	(0.71;1.31)	0.81	1.46^3^	(0.88;2.44)	0.14	***0.68***	***(0.43;1.06)***	***0.086***
*VDR*_2_: AAG (0,1,2)		1.25	(0.86;1.83)	0.25	0.87	(0.42;1.80)	0.71	***1.60***	***(0.97;2.63)***	***0.063***
*VDR*_3_: GAA (0,1,2)		1.13	(0.75;1.68)	0.56	0.69^3^	(0.32;1.50)	0.35	1.49	(0.89;2.49)	0.13
*VDR*_4_: AAA(0,1,2)		0.60	(0.31;1.14)	0.12	0.72	(0.26;1.95)	0.51	0.52	(0.21;1.28)	0.16
*MEGALIN*_1_: GCC (0,1,2)		0.75	(0.50;1.11)	0.15	0.70	(0.34;1.44)	0.33	0.72	(0.43;1.21)	0.22
*MEGALIN*_2_: ACC (0,1,2)		1.08	(0.74;1.58)	0.68	1.07	(0.55;2.12)	0.83	1.17	(0.71;1.92)	0.54

*Abbreviations*: BMI = body mass index (calculated as weight in kg/square of height in meters); SNP = Single Nucleotide polymorphism; SNPHAP = SNP haplotype; V2 = Visit 2; *VDR* = Vitamin D receptor gene; Note that *VDR*_1_*, VDR*_2_*, VDR*_3_ denote *VDR* SNPHAP, whereas *MEGALIN*_1_, *MEGALIN*_2_ and *MEGALIN*_3_ denote *MEGALIN* SNPHAP.

^1^See Table 1 for more details on definition the SNP haplotypes. (0,1,2) refers to ordinal coding with “0”, “1” and “2” copies of each haplotype. Three *VDR* SNP were combined to form the haplotypes, namely BsmI, ApaI and TaqI. Only haplotypes 1 and 2 were selected for *MEGALIN* since their overall prevalence was > 10%.

^2^Models were adjusted for age, sex, poverty status, education, current smoking status, current illicit drug use, self-rated health, total energy intake, HEI-2010 total score, 10 principal components for population structure, and the inverse mills ratio.

^3^P < 0.05 for null hypothesis that sex × SNPHAP interaction term = 0 in a model where main effect of sex was added.

Table S2 presents supplemental results for single SNP analyses for *VDR* and *MEGALIN* in relation to MetS outcomes, stratifying by gender. For incident MetS, overall, 15 SNP passed correction for multiple testing, of which one had a p = 0.001 (*MEGALIN* SNP: rs148386284: T allele, HR = 2.63, 95% CI:1.45–4.76). While many other SNPs were also associated with incident MetS, the ones that had p ≤ 0.01 were among women, including *MEGALIN* SNP rs830966: G allele, HR = 4.28, 95% CI:1.80–10.20) and 6 protective *VDR* SNPs located near a well-studied SNP names Cdx-I, rs11568250, whose C allele was also inversely related to incident MetS among women (HR = 0.11, 95%CI: 0.02–0.47, p = 0.003). Similarly, both *VDR* and *MEGALIN* single SNPs had significant associations with baseline and follow-up MetS outcomes, overall and among men and women, with few SNPs overlapping or being highly correlated with those that affected the incident outcome (e.g. incident MetS vs. follow-up MetS in women: rs830966-rs830969, rs2107301). Moreover, among the selected SNPs for VDR haplotypes, rs731236 (TaqI G > A) was associated with a reduced odds of baseline MetS (OR = 0.69, 95% CI: 0.50, 0.96, p = 0.029). Similarly, rs1544410 (BsmI G > A) was linked to an increased odds of baseline MetS among men (OR = 1.73, 95% CI: 1.01, 3.00, p = 0.045).

## Discussion

This study examined associations of selected haplotypes from SNPs in four *VDR* [rs1544410(BsmI:G/A); rs7975232(ApaI:A/C); rs731236(TaqI:G/A)], and two *MEGALIN* [rs3755166:G/A; rs2075252:C/T; rs2228171:C/T] gene haplotypes with longitudinal (n = 294–466) and cross-sectional (n = 539) metabolic outcomes among African Americans over a mean period of ~5 y of follow-up. Among key findings, in men, incident hypertension and MetS were inversely related to *MEGALIN*_1_ (GCC), and in women, *MEGALIN*_2_ (ACC) was consistently inversely related to incident hypertension and incident hyperglycemia. Among men, incident hyperglycemia was positively associated with *VDR*_1_ (GCA:baT), and among women, *VDR*_2_ (AAG:BAt) was directly associated with incident hypertension and *VDR*_3_ (GAA:Bat) was inversely related to incident central obesity. Overall, there was a direct, linear dose-response association between VDR_2_ (AAG:BAt), obesity and MetS at baseline. Moreover, *VDR*_3_ (GAA:bAT) was inversely related to baseline MetS among men, which differed significantly by sex. No associations were detected between *MEGALIN* haplotypes and outcomes at baseline or follow-up and 3 associations survived correction for multiple testing [*VDR*_2_ vs. baseline MetS (overall), *VDR*_3_ vs. baseline MetS (men) and *MEGALIN*_1_ vs. incident hypertension in men].

Recent studies with cross-sectional or case-control design have examined *VDR* polymorphisms as risk markers for central adiposity and related metabolic disorders^6,14–23^. However, none of these studies included African-American adults in their samples. When examining *VDR* SNP relationships with adiposity, a recent cross-sectional study (176 randomly selected men aged 25–65 y) found that homozygous *BsmI* (BB: AA *vs*. GG) was associated with higher BMI (29.0 *vs*. 26.8 kg/m^2^, p = 0.024) and higher WC (101.8 vs. 96.2 cm, p = 0.014)^15^. A similar finding was observed in another cross-sectional study of 153 women among whom body weight and fat mass were positively associated with the “BB” genotype of VDR SNP BsmI^16^. Similarly, in a third cross-sectional study, an association was found between a homozygous rare variant of rs3782905 located in the 3‘ *VDR* region (LD of rs3782905 with BsmI in White Hapmap is ~0.42) and 4.4 cm larger mean WC when compared with the homozygous common variant (Bonferroni-adjusted p = 0.004)^6^. These consistent findings for BsmI “A” allele dosage increasing the risk for obesity, were replicated with other related phenotypes, including T2D, fasting glucose level, LDL-Cholesterol and coronary heart disease risk in recent studies^17–20^. Using data from Baltimore Longitudinal Study of Aging, a previous study found that only the *Apa*I SNP (“C” allele dosage) significantly increased the odds of higher waist-to-hip ratio over time (*P*-trend = 0.024)^14^. These findings are consistent with ours, given that the “B” allele of BsmI corresponds to the “A” risk allele. In fact, findings from our present study indicated a positive association between MetS and the BAt *VDR* haplotype (i.e. *VDR*_2_) in the overall population and an inverse relationship with the bAT (i.e. *VDR*_3_) haplotype among men. Many other key results not surviving correction for multiple testing were generally trending in that same direction for various metabolic outcomes. Moreover, *MEGALIN* polymorphisms influenced central adiposity in Whites residing in Baltimore city: rs2075252 “TT” was associated with elevated waist to hip ratio at one point in time compared with rs2075252 “CC”^14^. Our finding with *MEGALIN*1 (GCC for rs3755166:G/A; rs2075252:C/T and rs2228171:C/T) being inversely related to incident hypertension indicates that in fact, a “C” allele for the middle SNP (rs2075252:C/T) may be protective against various obesity-related metabolic disturbance, particularly hypertension incidence rate. However, more studies are needed in diverse samples to replicate this finding.

Few previous studies have examined *VDR* SNPHAP as predictors of metabolic and cardiovascular outcomes. Despite earlier null findings, (e.g^21^.) more recent studies indicated that in fact BAt (*VDR*_2_) was associated with increased obesity risk, while the haplotype “GAG”, which was rare among our African-American urban adult population, was associated with a reduced risk of obesity^22^. Moreover, in a population-based study of men and women aged 55–80 y, each baT haplotype copy was associated with a 20% increased likelihood of ECG-confirmed myocardial infarction, adjusting for key confounders^23^. Similarly, in the BLSA study, an increased risk of longitudinal increase in WC among White women was uncovered with each baT haplotype copy^14^. In our present study, incident hyperglycemia risk was positively associated with baT, particularly among African-American men. Nevertheless, BAt, a less common haplotype in this population, was associated with a greater risk of incident hypertension in African-American women and with a greater likelihood of prevalent obesity and baseline MetS in the total African-American urban population. The latter finding (i.e. VDR_2_ vs. baseline MetS) was the only one that survived correction for multiple testing in the total population.

In terms of biological mechanisms, a greater *VDR* expression in adipocytes decreases energy expenditure markedly leading to increased adiposity. Further, VDR agonists reduce pro-inflammatory cytokines and D3 reduces high-glucose and LPS-induced TNFα and TGFβ release, suggesting a protective mechanism^22^. Moreover, high BMI is associated with low circulating 25(OH)D due to sequestration in adipose tissue^43^. Importantly, longer *VDR* BsmI polyA repeats exhibited less stability and translated less efficiently into VDR protein, resulting in a decreased vitamin D response, muscle cell inhibition and adipocyte differentiation^16^. In addition to the association of calcium and high Vitamin D intakes, vitamin D may also reduce hepatic synthesis of triglycerides and upregulate adiponectin expression, which in turn could reduce obesity and related metabolic disorders^44^. In the African-American population, *VDR* can trigger adiposity by modulating *VDR*-dependent molecular components of adipogenesis such as PPAR-γ and EBPα and thus inhibiting corresponding adipocyte differentiation^45^. Further, *VDR* variants may directly influence the binding of vitamin D and mediate various downstream effects on genes known to be VDR-responsive, thus influencing associated phenotypes^6^. Particularly for T2D, VDR may act as a transcription factor for $\beta$ cell insulin secretion regulation, thereby affecting lipid metabolism^46^.

Our study has several strengths, which include its longitudinal follow-up design, a large sample of a diverse urban population, and the extensive use of advanced statistical methods, through the combination of survival analysis, logistic regression, haplotype analysis and adjustment for selection biases.

Nevertheless, our study has notable limitations. First, our final analytic sample was likely selected in a non-random manner, whereby certain groups were over-sampled when compared with the original African-American sample in HANDLS. A 2-stage Heckman selection model was used to diminish those incurred biases^38^. Second, first-vist age and between-visit duration varied across participants, which may incur some imbalance in the data structure. Survival analysis methods were used to adjust for this imbalance. Moreover, our study had limited power to examine gene-environment interaction, particularly with 25(OH)D in serum or dietary intakes of vitamin D. Finally, positive results may have been chance findings, while negative findings may have been caused by lack of adequate power.

In conclusion, *VDR* and *MEGALIN* gene variations can affect the prevalence of MetS and the incidence of hypertension in a sex-specific manner, respectively, among African-American urban adults. Those study findings provide novel insights into the genetic variants at those gene loci and their association with susceptibility to cardiometabolic risk, including the metabolic syndrome among populations of African descent. Further functional studies of *VDR* and *MEGALIN* gene in relation to cardiometabolic risk can provide important validation for our results and can contribute to our understanding of how vitamin D metabolism-related genes can influence metabolic disorders in various populations. In addition, our findings if replicated by others can establish the need for a genetic screening test for *VDR* and *MEGALIN* polymorphisms. Thus, further large epidemiologic studies of similar populations are required to replicate our current findings.

## Electronic supplementary material

Supplemental methods 1 and 2
