# Supplementary material for: Vitamin D Metabolism-Related Gene Haplotypes and Their Association with Metabolic Disturbances Among African-American Urban Adults
Source: Sci Rep. 2018 May 23;8:8035. doi: 10.1038/s41598-018-26230-w (PMC5966433; doi:10.1038/s41598-018-26230-w)
Supplement: Supplementary file 1 — Supplemental methods 1 and 2 [file 41598_2018_26230_MOESM1_ESM.pdf]

VITAMIN D METABOLISM-RELATED GENE HAPLOTYPES AND THEIR ASSOCIATION WITH METABOLIC DISTURBANCES AMONG AFRICAN-AMERICAN URBAN ADULTS

May A. Beydoun[1,*]; Sharmin Hossain[1]; Salman M. Tajuddin[1]; Jose A. Canas[2]; Marie Kuczmarski[3]; Hind A. Beydoun[4]; Michele K. Evans[1§]; Alan B. Zonderman[1§]

[1] Laboratory of Epidemiology and Population Sciences, National Institute on Aging, NIA/NIH/IRP, Baltimore, MD.

[2] Pediatric Endocrinology, Nemours Children's Clinic, Jacksonville, FL.

[3]Department of Behavioral Health and Nutrition, University of Delaware, Newark, DE.

[4] Department of Medicine, Johns Hopkins School of Medicine, Baltimore, MD.

Supplemental Methods 1: Genetic data quality control

Sample quality control inclusion criteria were: **(1)** concordance between self-reported sex and X-chromosome estimated sex; **(2)** sample call rate >95%, **(3)** concordance between self-reported African ancestry and ancestry estimated using genotyped SNPs, and **(4)** proportional sharing of genotypes < 15% between samples, excluding close relatives from the final sample. SNPs in HANDLS were selected when the following criteria were met: **(1)** Hardy-Weinberg equilibrium p-value (HWE $P > 10^{-7}$); **(2)** Missing by haplotype $P > 10^{-7}$; **(3)** Minor allele frequency>0.01, and **(4)** SNP call rate >95%. Quality control and data management for each genotype was conducted using PLINKv1.06.[1] Cryptic relatedness was estimated via pairwise identity by descent analyses in PLINK and confirmed using RELPAIR.[2] STRUCTUREv2.3 [3-5] and multidimensional scaling (MDS) function in PLINKv1.06 were applied to determine ancestry among HANDLS participants. HANDLS participants with component vector estimates consistent with the HapMap African ancestry samples for the first 4 component vectors were included. Moreover, in a sensitivity analysis, we adjusted for all the first 10 principal components obtained from genotype data with MDS to control for residual effects of population structure.[6]. SNPs that passed quality control criteria were used for genotype imputation with MACH and minimac software (http://www.sph.umich.edu/csg/abecasis/mach/). The 1000 Genomes Project phase 1 alpha freeze multiethnic panel were used as a reference population for genotype imputation. SNPs with imputation quality measure of R^2<0.3 or minor allele frequency of <1% were excluded from further analyses.

Gene	SNP	Allele1	Allele2	Minor allele frequency (MAF)	Genotyped or imputed	Genotype call rate	R-square
VDR	rs731236	A	G	0.28034	Genotyped	0.996	-
VDR	rs7975232	C	A	0.37811	Genotyped	0.999	-
VDR	rs1544410	C	T	0.29485	Genotyped	0.999	-
MEGALIN	rs2075252	C	T	0.10177	Genotyped	0.998	-
MEGALIN	rs2228171	C	T	0.20141	Imputed	-	0.991
MEGALIN	rs3755166	G	A	0.3019	Genotyped	0.999	-

References

1. Purcell S, Neale B, Todd-Brown K, Thomas L, Ferreira MA, Bender D *et al.* PLINK: a tool set for whole-genome association and population-based linkage analyses. *Am J Hum Genet* 2007; **81**(3)**:** 559-75.

2. Epstein MP, Duren WL, Boehnke M. Improved inference of relationship for pairs of individuals. *Am J Hum Genet* 2000; **67**(5)**:** 1219-31.

3. Pritchard JK, Stephens M, Donnelly P. Inference of population structure using multilocus genotype data. *Genetics* 2000; **155**(2)**:** 945-59.

4. Falush D, Stephens M, Pritchard JK. Inference of population structure using multilocus genotype data: linked loci and correlated allele frequencies. *Genetics* 2003; **164**(4)**:** 1567-87.

5. Falush D, Stephens M, Pritchard JK. Inference of population structure using multilocus genotype data: dominant markers and null alleles. *Mol Ecol Notes* 2007; **7**(4)**:** 574-578.

6. Price AL, Patterson NJ, Plenge RM, Weinblatt ME, Shadick NA, Reich D. Principal components analysis corrects for stratification in genome-wide association studies. *Nat Genet* 2006; **38**(8)**:** 904-9.

Supplemental Methods 2: Single SNP analysis for all available high quality polymorphisms on the VDR and MEGALIN genes region.

758 SNPs in and around VDR and MEGALIN genes were found in the HANDLS database, that were of good quality based on the same criteria described in supplemental method 1. A secondary analysis was conducted whereby each SNP was entered into the same models as for SNPHAPs along with the same set of covariates and the same series of incident and prevalent metabolic syndrome outcomes, stratifying by sex. Effect modification by sex was also tested. The critical p-value was set at 0.01 for overall analysis and 0.02 for sex-specific analyses. Only statistically significant findings after correction for multiple testing are presented. Noteworthy results with p≤0.001 are highlighted. Below is the complete list of SNPs included in the analyses, along with the allele dosage definition based on Al1, and Mean Allele Frequency of Al1. CHR = 2 for *MEGALIN* and CHR=12 for *VDR*.

TABLE S1. Selected SNPs for supplemental analysis

CHR	SNP	Al1	Al2	MAF
2	rs7566044	A	G	0.2529
2	rs72888673	A	G	0.07764
2	rs79004576	A	G	0.0498
2	rs12328794	T	C	0.2373
2	rs12105342	T	C	0.2012
2	rs3815574	A	C	0.2168
2	rs2302698	A	G	0.2026
2	rs736020	T	C	0.2026
2	rs10192264	T	C	0.2993

2	rs4668119	G	A	0.3052
2	rs6750251	G	C	0.4097
2	rs12474309	C	G	0.1299
2	rs75950705	A	T	0.07959
2	rs62172561	C	T	0.03564
2	rs4668120	C	G	0.4834
2	rs75825993	C	G	0.1274
2	rs7606912	G	A	0.4023
2	rs79890698	T	G	0.1416
2	rs4667590	G	C	0.3423
2	rs75180829	C	T	0.1436
2	rs1861609	G	A	0.2476
2	rs2389598	T	C	0.4932
2	rs113349208	G	A	0.1431
2	rs6433105	T	C	0.2334
2	rs2111187	C	T	0.4907
2	rs4668121	T	G	0.4331
2	rs73028985	A	G	0.1084
2	rs1990702	T	C	0.1821
2	rs7574148	C	T	0.01904
2	rs73028988	T	C	0.1084
2	rs73028989	C	A	0.1089
2	rs10191750	A	G	0.4102
2	rs6433106	T	C	0.2808
2	rs6433107	T	G	0.1821
2	rs78597444	A	G	0.01123
2	rs78655335	T	A	0.1089
2	rs12466193	T	G	0.1836

2	rs12478045	C	G	0.3198
2	rs13406401	A	G	0.3711
2	rs55670659	T	C	0.4028
2	rs11884346	C	T	0.3408
2	rs73028996	G	A	0.1108
2	rs7598209	T	C	0.1685
2	rs1003456	A	T	0.1704
2	rs73030803	G	T	0.1094
2	rs6754932	C	T	0.1895
2	rs11691854	T	C	0.3911
2	rs139023066	G	C	0.3823
2	rs150914001	A	C	0.2964
2	rs144260784	G	A	0.1069
2	rs115648286	C	T	0.3447
2	rs58389457	G	A	0.333
2	rs7576280	G	A	0.1792
2	rs11693676	G	C	0.1138
2	rs139334150	C	T	0.2314
2	rs11892075	A	G	0.3906
2	rs140706266	T	C	0.1084
2	rs4264540	T	G	0.2344
2	rs11674531	G	T	0.4512
2	rs6746604	C	G	0.314
2	rs990627	T	C	0.312
2	rs990626	A	G	0.312
2	rs73970130	C	A	0.1826
2	rs2268380	A	G	0.3125
2	rs2268379	G	A	0.4561

2	rs2268378	A	G	0.4346
2	rs6725805	G	A	0.4385
2	rs16856488	G	A	0.2114
2	rs2075253	G	A	0.249
2	rs6733111	G	A	0.293
2	rs6733122	A	G	0.3672
2	rs146901930	A	G	0.08398
2	rs2284681	C	A	0.3672
2	rs68155726	T	G	0.3345
2	rs2284680	C	A	0.3711
2	rs4668122	A	G	0.4014
2	rs2239592	C	T	0.29
2	rs2239591	T	C	0.3701
2	rs2239590	A	T	0.3701
2	rs2239589	G	T	0.3706
2	rs4667591	G	T	0.2246
2	rs41268687	A	G	0.209
2	rs4667592	G	A	0.4736
2	rs55681838	C	T	0.05029
2	rs6761244	A	G	0.4517
2	rs1123904	A	C	0.4087
2	rs1123905	T	C	0.4087
2	rs12692892	G	A	0.4497
2	rs10192078	T	C	0.4541
2	rs741376	A	C	0.4087
2	rs72874715	T	A	0.06104
2	rs755631	C	A	0.4844
2	rs17848195	A	G	0.1865

2	rs3944004	C	A	0.2832
2	rs4667593	A	G	0.4102
2	rs4667594	T	A	0.4014
2	rs11679947	G	A	0.4097
2	rs10490132	C	A	0.4097
**2**	**rs2075252**	**T**	**C**	**0.1021**
2	rs2075251	T	A	0.1387
2	rs3815573	A	G	0.377
2	rs13021137	G	T	0.4941
2	rs6759013	A	G	0.3882
2	rs6730825	C	T	0.4863
2	rs13011165	T	C	0.3882
2	rs13034796	G	A	0.376
2	rs2268377	C	A	0.4624
2	rs2268376	T	C	0.3882
2	rs9646777	G	A	0.4932
2	rs9646778	T	C	0.3882
2	rs3821124	T	G	0.4624
2	rs2268375	G	A	0.4546
2	rs2268374	G	T	0.1021
2	rs11884342	A	G	0.4595
2	rs11896574	T	A	0.4272
2	rs2229268	G	A	0.05176
2	rs2239602	C	T	0.05078
2	rs2239601	A	G	0.2515
2	rs4140872	C	T	0.1011
2	rs7559094	A	T	0.25
2	rs67931300	C	T	0.1875

2	rs4497843	A	G	0.2212
2	rs17848190	C	T	0.1201
2	rs17848189	A	C	0.09033
2	rs2075250	C	T	0.09033
2	rs2024481	A	C	0.4092
2	rs2229265	T	C	0.3423
2	rs2193196	T	C	0.1938
2	rs7565788	T	C	0.2388
2	rs741378	G	A	0.4263
2	rs10191692	C	T	0.4053
2	rs7421492	C	T	0.4009
2	rs112597789	A	G	0.08398
2	rs2892803	C	T	0.4033
2	rs9789747	C	T	0.08838
2	rs9287910	C	T	0.3076
2	rs9287911	A	T	0.2368
2	rs6744473	T	A	0.3491
2	rs17848180	T	C	0.04297
2	rs3213759	T	G	0.2158
2	rs10169879	T	C	0.2437
2	rs10204688	C	T	0.1885
2	rs7588584	T	C	0.2412
2	rs2024480	A	G	0.1885
2	rs2284679	C	T	0.2495
2	rs6744155	G	A	0.2412
2	rs2284678	A	G	0.2324
2	rs2284677	G	A	0.2412
2	rs2284676	G	A	0.3203

2	rs4287730	G	C	0.2188
2	rs6747214	C	T	0.2524
2	rs59363833	C	T	0.2793
2	rs16856558	A	G	0.2397
2	rs10210408	T	C	0.4229
2	rs1548936	C	T	0.2002
2	rs1972589	C	T	0.2612
2	rs57926641	C	T	0.2783
2	rs7565822	G	A	0.4819
2	rs7592152	A	C	0.08936
2	rs16856573	T	C	0.2031
2	rs4331469	A	C	0.4736
2	rs2389589	A	G	0.2695
2	rs3770604	T	A	0.2715
2	rs13388593	T	C	0.2349
2	rs3821125	T	C	0.4834
2	rs2075248	T	C	0.1919
2	rs73971311	T	G	0.1875
2	rs11886626	T	C	0.2036
2	rs2075247	T	C	0.3867
2	rs10490131	G	A	0.03174
2	rs58687448	C	T	0.2007
2	rs77463292	T	C	0.03174
2	rs7557964	C	T	0.2949
2	rs1972588	T	G	0.008789
2	rs7578722	C	T	0.1963
**2**	**rs2228171**	**T**	**C**	**0.2007**
2	rs2302696	T	C	0.4297

2	rs73033887	T	C	0.2764
2	rs10169232	C	G	0.2842
2	rs16856592	C	A	0.2803
2	rs16856593	A	G	0.271
2	rs16856596	A	G	0.1992
2	rs62172607	A	G	0.3027
2	rs62172609	A	G	0.3022
2	rs16823023	C	T	0.1992
2	rs11898106	G	A	0.2764
2	rs73033899	T	C	0.27
2	rs73971315	G	T	0.2993
2	rs28454851	A	G	0.3779
2	rs77726104	C	T	0.1313
2	rs11687903	G	T	0.4277
2	rs13401581	A	G	0.3779
2	rs79399342	G	T	0.1318
2	rs16856600	A	G	0.2031
2	rs2239600	T	C	0.4268
2	rs2284675	A	G	0.4268
2	rs2239599	C	T	0.4302
2	rs6725137	T	C	0.3003
2	rs13410285	A	T	0.249
2	rs13397109	C	G	0.2993
2	rs35114151	G	A	0.1055
2	rs13417389	C	T	0.3052
2	rs2268373	G	C	0.3535
2	rs2268372	A	T	0.3276
2	rs10200740	C	T	0.3633

2	rs10200859	C	T	0.3535
2	rs10188487	T	C	0.3276
2	rs79503405	T	G	0.2705
2	rs62172612	C	G	0.09521
2	rs75569504	G	A	0.3496
2	rs116247504	C	T	0.2417
2	rs112172369	C	G	0.2847
2	rs10170902	G	A	0.1704
2	rs4668124	A	C	0.07031
2	rs77711606	C	T	0.2085
2	rs4001547	G	C	0.06982
2	rs11689553	G	C	0.05273
2	rs10201691	A	G	0.2734
2	rs13422498	T	G	0.1709
2	rs10201911	T	C	0.2012
2	rs6718884	C	T	0.4941
2	rs11886219	C	T	0.4175
2	rs2302695	G	C	0.2764
2	rs75092581	A	G	0.1675
2	rs17848164	T	C	0.2959
2	rs2052298	A	T	0.3525
2	rs2052297	C	T	0.3521
2	rs10190812	T	C	0.2388
2	rs2052296	C	A	0.2368
2	rs62172631	A	G	0.3965
2	rs11897009	T	C	0.2383
2	rs11886185	T	A	0.2505
2	rs11886318	A	C	0.2754

2	rs13417486	T	C	0.2339
2	rs13431061	C	T	0.2842
2	rs62172632	T	G	0.2163
2	rs7600336	C	T	0.314
2	rs2300447	C	A	0.3716
2	rs2300446	T	C	0.3672
2	rs2193195	T	C	0.4736
2	rs2193194	G	A	0.3452
2	rs2193193	G	A	0.2749
2	rs2216239	C	T	0.3423
2	rs3815572	T	C	0.27
2	rs2268370	A	C	0.3115
2	rs9283479	C	T	0.3149
2	rs9646731	A	G	0.2646
2	rs7569236	T	C	0.2339
2	rs4606889	T	C	0.3149
2	rs4302191	C	G	0.2808
2	rs6719440	C	T	0.3149
2	rs6747692	T	A	0.3291
2	rs2268369	A	C	0.2822
2	rs2268368	T	C	0.3335
2	rs2268367	A	C	0.2822
2	rs2268366	A	T	0.3335
2	rs13389381	C	T	0.376
2	rs11902433	C	T	0.29
2	rs34951037	A	T	0.3208
2	rs2075246	A	G	0.2886
2	rs982810	A	G	0.2871

2	rs13383183	A	C	0.3799
2	rs35836996	T	A	0.3779
2	rs6433109	A	C	0.2764
2	rs3915725	A	G	0.2383
2	rs4668127	A	G	0.3643
2	rs4668128	G	A	0.2817
2	rs2302694	A	G	0.3848
2	rs2302693	T	C	0.2729
2	rs2302692	C	T	0.3574
2	rs3926693	T	C	0.3574
2	rs1816039	T	C	0.2388
2	rs4667597	A	G	0.2056
2	rs3821126	A	G	0.3164
2	rs1362996	G	A	0.4009
2	rs3821127	A	G	0.2817
2	rs3821128	T	C	0.2739
2	rs2239598	G	A	0.4453
2	rs2239597	C	A	0.3496
2	rs2239596	C	T	0.3877
2	rs2239595	A	G	0.03174
2	rs2239594	C	T	0.2778
2	rs6713797	A	T	0.3242
2	rs151020693	C	T	0.1318
2	rs6752778	A	C	0.3223
2	rs6724600	G	T	0.4556
2	rs13401167	G	A	0.1772
2	rs114534086	C	T	0.1338
2	rs4668129	G	A	0.3804

2	rs115452726	A	C	0.1357
2	rs148175287	A	G	0.1323
2	rs114658487	T	C	0.1323
2	rs2229267	A	G	0.3623
2	rs78967293	T	G	0.1323
2	rs78008770	A	T	0.1323
2	rs12987817	G	A	0.106
2	rs2268365	C	T	0.03174
2	rs78265059	T	C	0.1323
2	rs830973	A	G	0.3823
2	rs34915742	G	C	0.1323
2	rs76714416	G	A	0.1323
2	rs76838238	T	C	0.1323
2	rs2239593	A	G	0.08154
2	rs111495150	C	T	0.1323
2	rs12615180	A	C	0.008301
2	rs78828988	T	C	0.1318
2	rs77416334	G	T	0.1318
2	rs3755164	A	C	0.003418
2	rs10490130	C	A	0.1729
2	rs831040	C	T	0.4004
2	rs831041	T	G	0.312
2	rs831042	T	C	0.4019
2	rs2075254	G	A	0.312
2	rs56377101	A	G	0.2075
2	rs12613980	T	G	0.1504
2	rs831043	T	C	0.312
2	rs2075249	T	G	0.272

2	rs831044	A	T	0.3906
2	rs16823029	A	C	0.1836
2	rs831046	G	A	0.07764
2	rs830956	C	T	0.3438
2	rs830957	C	T	0.4297
2	rs830959	C	T	0.3438
2	rs830960	C	T	0.3438
2	rs1421509	T	C	0.3135
2	rs2241190	T	C	0.3203
2	rs33954745	G	A	0.144
2	rs35583956	T	A	0.1992
2	rs830982	G	A	0.3872
2	rs13025890	G	C	0.145
2	rs830983	A	G	0.395
2	rs12988804	T	C	0.1855
2	rs1096456	T	C	0.3174
2	rs138822865	A	C	0.06885
2	rs36198025	G	A	0.1724
2	rs141755776	T	C	0.006836
2	rs830989	A	G	0.3794
2	rs830991	T	C	0.3716
2	rs35994058	T	A	0.1714
2	rs68108873	C	T	0.1343
2	rs35853478	T	C	0.1372
2	rs2673175	T	G	0.165
2	rs4667599	G	A	0.4316
2	rs4613240	C	T	0.06885
2	rs2544386	A	T	0.2524

2	rs2544387	G	A	0.2827
2	rs12993779	T	C	0.1079
2	rs2544388	G	A	0.2822
2	rs148771995	C	G	0.01904
2	rs191510832	G	T	0.04102
2	rs139905197	T	G	0.3735
2	rs76668605	A	G	0.3447
2	rs74624697	T	G	0.2754
2	rs141248390	A	G	0.2476
2	rs6748227	C	T	0.269
2	rs6719945	A	G	0.3838
2	rs6706284	T	C	0.3667
2	rs6706290	T	C	0.3574
2	rs6706292	T	A	0.3477
2	rs143010438	T	C	0.06348
2	rs830992	A	G	0.3042
2	rs830993	A	T	0.2754
2	rs10515931	T	C	0.05811
2	rs830994	G	A	0.4658
2	rs830995	A	G	0.3477
2	rs10515930	C	G	0.06396
2	rs10490129	C	G	0.04395
2	rs77612812	A	C	0.04395
2	rs3770607	T	G	0.01904
2	rs830997	A	G	0.3364
2	rs830998	A	C	0.2173
2	rs830999	G	A	0.252
2	rs831000	T	C	0.2173

2	rs831001	T	C	0.2163
2	rs831002	T	C	0.2231
2	rs831003	G	C	0.2422
2	rs2673179	G	A	0.3105
2	rs2544372	C	T	0.2212
2	rs62173979	G	A	0.06055
2	rs7568568	C	T	0.1787
2	rs59076959	C	T	0.07178
2	rs62173981	G	A	0.4248
2	rs831004	T	C	0.3657
2	rs831005	G	A	0.4341
2	rs831006	C	G	0.3418
2	rs73037812	G	T	0.2334
2	rs831007	T	C	0.2827
2	rs831008	C	T	0.1729
2	rs831009	G	A	0.2646
2	rs831010	C	T	0.1841
2	rs72878449	G	A	0.2285
2	rs831011	A	G	0.2637
2	rs831012	C	T	0.2861
2	rs831013	T	G	0.3716
2	rs56325975	C	G	0.1338
2	rs75581025	G	A	0.06885
2	rs831014	T	G	0.1836
2	rs831015	T	C	0.3853
2	rs72878458	T	C	0.125
2	rs55679014	G	T	0.2031
2	rs58338106	T	C	0.2886

2	rs9653235	A	C	0.06641
2	rs11896551	T	C	0.07129
2	rs831016	G	C	0.4102
2	rs3770611	C	A	0.4077
2	rs3770612	G	A	0.1201
2	rs72878472	C	T	0.1616
2	rs10754970	A	G	0.2368
2	rs72878477	T	C	0.1064
2	rs2161039	T	C	0.252
2	rs831017	G	A	0.4648
2	rs16856748	A	G	0.1235
2	rs831019	T	G	0.4565
2	rs3770613	T	C	0.355
2	rs61219833	C	T	0.09521
2	rs831020	A	G	0.1929
2	rs59457398	C	T	0.09521
2	rs72878487	A	G	0.07275
2	rs62171263	A	G	0.1753
2	rs831022	C	T	0.4785
2	rs11887007	A	C	0.3472
2	rs62171264	A	G	0.01562
2	rs2229266	A	G	0.3062
2	rs16856759	G	A	0.374
2	rs2673165	T	C	0.395
2	rs13396247	A	T	0.2725
2	rs2673164	T	C	0.3965
2	rs2673163	C	T	0.1689
2	rs3770615	G	A	0.02393

2	rs2673162	T	C	0.3315
2	rs2222020	A	C	0.3652
2	rs2222019	A	C	0.3657
2	rs9287914	A	T	0.2861
2	rs2544373	T	A	0.375
2	rs2544374	G	A	0.2129
2	rs2244407	T	C	0.1694
2	rs830962	A	G	0.4658
2	rs830963	A	G	0.457
2	rs3770616	T	C	0.2632
2	rs7575260	A	G	0.2632
2	rs830964	C	T	0.4233
2	rs4667600	A	T	0.2666
2	rs3914468	G	A	0.2734
2	rs830965	A	G	0.4038
2	rs2892802	T	G	0.06738
2	rs700550	C	T	0.2036
2	rs830966	G	C	0.2593
2	rs830967	A	T	0.2041
2	rs830968	T	C	0.2041
2	rs830969	A	G	0.2075
2	rs58208595	T	C	0.01172
2	rs853988	C	A	0.2124
2	rs830970	T	C	0.2266
2	rs7600757	A	G	0.4966
2	rs830971	C	G	0.2266
2	rs830972	G	A	0.2124
2	rs12622085	G	A	0.1011

2	rs2247506	C	T	0.313
2	rs17848175	A	G	0.05078
2	rs2673170	C	G	0.4478
2	rs2673169	C	G	0.4541
2	rs80083165	C	T	0.4561
2	rs142912973	C	T	0.3677
2	rs6752012	A	C	0.4233
2	rs4638759	C	G	0.2959
2	rs7604111	A	G	0.4575
2	rs10177180	G	A	0.438
2	rs10177361	A	G	0.4365
2	rs3845730	G	A	0.4429
2	rs2544376	C	T	0.458
2	rs2544377	A	G	0.458
2	rs2673167	G	C	0.4648
2	rs2544378	T	C	0.4214
2	rs2544379	A	G	0.4214
2	rs2544380	T	G	0.3931
2	rs2544381	C	G	0.4438
2	rs10221870	A	C	0.3765
2	rs3770623	T	C	0.4336
2	rs3770624	C	A	0.436
2	rs2544383	T	A	0.4419
2	rs2544384	C	T	0.4419
2	rs861239	C	T	0.4355
2	rs2229263	C	T	0.4355
2	rs830974	C	T	0.4414
2	rs28490283	T	C	0.01318

2	rs830976	T	C	0.4365
2	rs830977	C	G	0.436
2	rs830979	A	G	0.4863
2	rs2673178	G	A	0.4409
2	rs2673177	A	G	0.4409
2	rs3845731	A	G	0.2705
2	rs13006076	G	T	0.01514
2	rs831025	A	T	0.4058
2	rs3770630	C	T	0.2583
2	rs6433115	C	T	0.2583
2	rs831027	T	C	0.08691
2	rs74505905	T	C	0.0498
2	rs831029	C	G	0.3872
2	rs78991977	G	A	0.07764
2	rs831030	T	C	0.4072
2	rs831031	G	A	0.4248
2	rs831032	A	G	0.1274
2	rs831034	T	C	0.1245
2	rs10930352	C	T	0.2759
2	rs11889511	G	C	0.2617
2	rs6751001	T	C	0.2773
2	rs60641214	T	A	0.3438
2	rs16856823	T	A	0.08105
2	rs831036	C	G	0.4307
2	rs831037	G	A	0.4424
2	rs831038	C	T	0.4424
2	rs3770636	G	T	0.08105
2	rs3770637	C	T	0.0708

2	rs3729573	T	C	0.4736
2	rs2673171	G	A	0.07568
2	rs77073020	G	A	0.1489
2	rs3821129	C	T	0.06641
2	rs2673172	G	T	0.3486
2	rs2544390	C	T	0.3486
2	rs2390793	T	C	0.2285
2	rs3770641	T	A	0.2065
2	rs6730118	G	A	0.2798
2	rs2544392	T	C	0.3862
2	rs4668134	T	A	0.08252
2	rs2389558	T	C	0.3198
2	rs4668135	A	C	0.06055
2	rs13002515	T	A	0.1382
2	rs13003297	C	G	0.1377
2	rs6713072	T	C	0.3164
2	rs1990842	C	A	0.2051
2	rs12614394	G	A	0.1372
2	rs10199321	T	C	0.2095
2	rs10199676	T	G	0.21
2	rs16856840	A	G	0.06836
2	rs2673151	T	C	0.1406
2	rs13017888	T	C	0.1377
2	rs13017872	G	A	0.1406
2	rs13017879	T	A	0.1377
2	rs6721930	C	G	0.2769
2	rs2389557	A	G	0.4673
2	rs830943	A	G	0.1465

2	rs12995288	G	A	0.1362
2	rs34038476	T	A	0.1372
2	rs16856843	A	G	0.0791
2	rs4668136	T	C	0.3218
2	rs3845732	C	T	0.438
2	rs12692895	T	C	0.1279
2	rs3815679	T	C	0.04736
2	rs3815680	G	T	0.08008
2	rs2161038	C	G	0.1382
**2**	**rs3755166**	**A**	**G**	**0.3018**
2	rs6755801	T	A	0.2974
2	rs12612683	C	T	0.1323
2	rs830952	C	A	0.4468
2	rs700552	C	G	0.4971
2	rs58599996	A	C	0.1782
2	rs830954	C	T	0.4932
2	rs830955	A	C	0.4927
2	rs10167272	C	T	0.1821
2	rs148386284	T	G	0.2554
2	rs112277758	C	G	0.2632
2	rs140297819	G	C	0.1743
2	rs145088245	C	T	0.05273
2	rs2544371	G	A	0.4243
2	rs2673166	C	A	0.2847
2	rs61707661	T	C	0.3359
2	rs143130711	C	T	0.04346
2	rs13026171	T	C	0.2573
2	rs2673159	C	T	0.4395

2	rs13002827	C	T	0.07715
2	rs12470146	A	C	0.008789
2	rs62171289	A	T	0.356
2	rs12616998	C	T	0.4062
2	rs12613663	T	G	0.4062
2	rs78893940	C	T	0.06396
2	rs12692897	G	A	0.251
2	rs12692898	G	A	0.4062
2	rs1344	G	A	0.4058
2	rs1356057	C	G	0.4048
2	rs1356056	C	T	0.4043
2	rs13423674	G	A	0.4043
2	rs12692899	A	C	0.4038
2	rs12692900	G	A	0.249
2	rs12622978	C	A	0.1562
2	rs7567342	C	T	0.4048
2	rs11900616	A	T	0.4058
2	rs11895060	A	C	0.4111
2	rs4667601	A	G	0.03027
2	rs4566330	G	A	0.4912
2	rs34155127	G	A	0.05811
2	rs4668139	G	A	0.05859
2	rs1606797	C	A	0.4824
2	rs6721866	T	C	0.1528
2	rs4338933	T	C	0.2798
2	rs12463500	G	A	0.1343
2	rs34996026	T	C	0.1353
2	rs4668141	C	T	0.4478

12	rs11168262	T	C	0.1206
12	rs12721364	A	G	0.03271
12	rs7965281	G	A	0.375
12	rs10783215	C	T	0.4014
12	rs7968585	C	T	0.3965
12	rs2525046	T	C	0.251
12	rs2408875	T	G	0.4976
12	rs11574143	T	C	0.08789
12	rs2853563	T	C	0.1597
12	rs2853562	T	A	0.3052
12	rs9729	G	T	0.4292
12	rs3847987	A	C	0.07373
12	rs739837	G	T	0.4331
**12**	**rs731236**	**G**	**A**	**0.2803**
**12**	**rs7975232**	**C**	**A**	**0.3779**
12	rs11574114	T	C	0.1587
12	rs11574113	G	C	0.08398
12	rs10875692	T	C	0.005371
12	rs757343	T	C	0.07373
**12**	**rs1544410**	**T**	**C**	**0.2944**
12	rs55748765	T	C	0.3281
12	rs10783217	A	G	0.2197
12	rs2238141	C	T	0.4238
12	rs2525044	A	G	0.2222
12	rs7139204	G	C	0.4233
12	rs12314197	G	A	0.2051
12	rs7962898	T	C	0.3623
12	rs58789572	T	C	0.2007

12	rs7963776	G	A	0.4238
12	rs4760732	C	T	0.4751
12	rs4760733	A	G	0.4502
12	rs7967152	A	C	0.3979
12	rs2239185	G	A	0.4233
12	rs2239184	G	A	0.3809
12	rs7971418	C	A	0.4512
12	rs7975128	A	G	0.2798
12	rs11168264	G	A	0.2129
12	rs113322950	C	T	0.2471
12	rs7296204	G	A	0.2393
12	rs7316602	C	T	0.271
12	rs11168265	T	C	0.2832
12	rs7305032	G	A	0.2886
12	rs11168266	C	T	0.4111
12	rs11168267	A	G	0.07227
12	rs11168268	G	A	0.3589
12	rs2238140	G	A	0.4907
12	rs2248098	A	G	0.4937
12	rs2283344	T	C	0.3208
12	rs12370156	C	T	0.4883
12	rs987849	G	A	0.2529
12	rs2283343	A	G	0.4668
12	rs2239182	T	C	0.4058
12	rs2107301	A	G	0.166
12	rs2283342	G	A	0.02783
12	rs2239181	C	A	0.1323
12	rs2239180	G	C	0.1318

12	rs2238139	G	A	0.2378
12	rs1540339	T	C	0.228
12	rs2239179	C	T	0.334
12	rs12717991	T	C	0.3091
12	rs7965360	A	G	0.3647
12	rs7968852	A	G	0.292
12	rs7308350	A	C	0.3174
12	rs12721370	A	C	0.07422
12	rs886441	G	A	0.4111
12	rs1808208	G	A	0.2461
12	rs73109883	A	G	0.1152
12	rs2189480	T	G	0.3589
12	rs2238138	A	G	0.2271
12	rs2238137	T	C	0.03711
12	rs12721395	A	T	0.1826
12	rs59707231	T	A	0.1826
12	rs3819545	G	A	0.2583
12	rs117572434	C	T	0.1895
12	rs12721396	A	G	0.228
12	rs3782905	C	G	0.2192
12	rs61919100	G	C	0.04102
12	rs113270938	T	C	0.04102
12	rs7311713	T	G	0.231
12	rs11168274	T	C	0.3232
12	rs181665806	T	G	0.1226
12	rs12721397	G	A	0.2295
12	rs2239186	G	A	0.05566
12	rs10875693	A	T	0.167

12	rs7974353	T	C	0.1313
12	rs61919101	G	A	0.1011
12	rs7974708	C	T	0.1997
12	rs6580642	T	C	0.1338
12	rs11168275	C	T	0.3262
12	rs11574050	A	G	0.06104
12	rs10783218	A	G	0.1318
12	rs2228570	A	G	0.2188
12	rs2408876	C	T	0.4482
12	rs2254210	A	G	0.3237
12	rs2408877	T	A	0.08984
12	rs7297462	C	T	0.07568
12	rs12721373	A	T	0.01953
12	rs11574044	C	A	0.2021
12	rs11574042	G	C	0.1255
12	rs2238136	T	C	0.08545
12	rs1989969	A	G	0.4316
12	rs2238135	G	C	0.293
12	rs2853564	G	A	0.1338
12	rs7965266	T	A	0.1313
12	rs7965274	T	C	0.1313
12	rs12321826	T	C	0.09668
12	rs7979131	G	T	0.1313
12	rs4760648	C	T	0.4565
12	rs4760649	A	G	0.1978
12	rs2853561	T	C	0.332
12	rs10875694	A	T	0.09375
12	rs12298585	G	C	0.127

12	rs11168283	T	C	0.1875
12	rs2853559	A	G	0.1909
12	rs12721375	A	G	0.02344
12	rs11168284	G	A	0.4355
12	rs7965943	T	G	0.3882
12	rs2853566	G	A	0.189
12	rs2853565	G	A	0.2646
12	rs11168286	A	G	0.03467
12	rs4760650	T	G	0.4883
12	rs7966244	T	A	0.417
12	rs12302580	C	G	0.2158
12	rs3922882	G	C	0.1611
12	rs11168287	G	A	0.3042
12	rs4328262	G	T	0.3232
12	rs4334089	G	A	0.4009
12	rs4237855	G	A	0.2612
12	rs5013378	G	A	0.4985
12	rs4341603	G	T	0.4912
12	rs11574027	A	C	0.01318
12	rs7965397	G	T	0.1143
12	rs3890734	A	G	0.1479
12	rs3890733	T	C	0.1489
12	rs111336890	C	T	0.1196
12	rs7302235	C	T	0.4829
12	rs58379944	G	A	0.09717
12	rs10875695	A	C	0.4829
12	rs11168292	G	C	0.1479
12	rs11168293	T	G	0.1479

12	rs4760655	G	A	0.08105
12	rs7136534	T	C	0.09229
12	rs12581281	T	C	0.03174
12	rs10783219	T	A	0.08008
12	rs10083198	T	C	0.2524
12	rs7299460	C	T	0.25
12	rs4760658	G	A	0.1514
12	rs7979360	G	A	0.4434
12	rs11574012	G	A	0.03369
12	rs4516035	C	T	0.08203
12	rs7139166	G	C	0.08203
12	rs10875696	T	G	0.09082
12	rs11614332	A	G	0.09277
**12**	**rs11568820**	**C**	**T**	**0.21**
12	rs11168297	A	G	0.08447
12	rs4760603	T	A	0.08301
12	rs7310552	G	A	0.08398
12	rs4411327	C	T	0.4399
12	rs7975847	T	C	0.4404
12	rs7976091	C	T	0.2104
12	rs10875697	T	A	0.3535

Note: Bolded SNPs are the ones that were selected for haplotype analysis.

Table S2. Key findings from single SNP analysis with incident and prevalent MetS outcomes, overall and stratified by sex.

**********************Incident MetS*****************

<u>Overall</u>:

SNPrs11898106_g		2.292095	.7141345	2.66	0.008	1.244597	4.221205
SNPrs830969_a		2.445877	.8414834	2.60	0.009	1.246194	4.800465
SNPrs853988_c		2.591791	.9185341	2.69	0.007	1.293996	5.19119
SNPrs830972_g		2.591791	.9185341	2.69	0.007	1.293996	5.19119
SNPrs17848175_a		3.519254	1.57947	2.80	0.005	1.46024	8.481584
SNPrs831032_a		3.045186	1.1736	2.89	0.004	1.430753	6.481311
SNPrs831034_t		3.106713	1.200571	2.93	0.003	1.456664	6.625869
SNPrs3815679_t		4.828003	2.819711	2.70	0.007	1.536873	15.16691
**SNPrs148386284_t**		**2.626265**	**.796895**	**3.18**	**0.001**	**1.448959**	**4.760155**
SNPrs112277758_c		2.568595	.7721236	3.14	0.002	1.425027	4.629863
SNPrs2544371_g		.4211706	.1372794	-2.65	0.008	.2223368	.7978198
SNPrs13026171_t		2.502543	.7536467	3.05	0.002	1.386885	4.515678
SNPrs2673159_c		.457887	.1367192	-2.62	0.009	.2550355	.8220839
SNPrs12692897_g		2.58272	.8294801	2.95	0.003	1.376261	4.846785
SNPrs12692900_g		2.379948	.7512276	2.75	0.006	1.281996	4.418228

<u>Men</u>

SNPrs75950705_a		154.1337	262.3659	2.96	0.003	5.482585	4333.213
SNPrs10204688_c		31.90083	47.09618	2.35	0.019	1.766634	576.046
SNPrs2024480_a		31.90083	47.09618	2.35	0.019	1.766634	576.046
SNPrs16856573_t		.0067755	.0124439	-2.72	0.007	.0001852	.2478918
SNPrs2302696_t		29.30606	38.48466	2.57	0.010	2.2344	384.3738
SNPrs2052297_c		203.4858	398.0846	2.72	0.007	4.398515	9413.737
SNPrs10190812_t		.0259778	.0401426	-2.36	0.018	.0012568	.5369498
SNPrs11897009_t		.0259778	.0401426	-2.36	0.018	.0012568	.5369498
SNPrs4668128_g		18.97355	22.73207	2.46	0.014	1.812717	198.5944
SNPrs2239594_c		23.1881	30.39854	2.40	0.016	1.775737	302.797
SNPrs6724600_g		155.2049	295.0959	2.65	0.008	3.736706	6446.467
SNPrs2673175_t		.0001106	.000427	-2.36	0.018	5.74e-08	.2133398
SNPrs6706284_t		.028124	.0395078	-2.54	0.011	.001792	.4413881
SNPrs6706292_t		.003089	.0075579	-2.36	0.018	.0000255	.3736632
SNPrs830994_g		.0187236	.0308215	-2.42	0.016	.0007434	.471608
SNPrs2161039_t		1008.545	2775.499	2.51	0.012	4.583552	221915.8
SNPrs62171263_a		1454.726	4152.921	2.55	0.011	5.404916	391537.5
SNPrs2222020_a		.0370027	.0461541	-2.64	0.008	.0032101	.4265327
SNPrs2222019_a		.0370027	.0461541	-2.64	0.008	.0032101	.4265327
SNPrs9287914_a		17.46258	20.51715	2.43	0.015	1.745879	174.6637
SNPrs2544374_g		.0639913	.0742439	-2.37	0.018	.0065848	.6218727

SNPrs2544373_t	.0370031	.0461548	-2.64	0.008	.0032101	.4265411
SNPrs4667600_a	10.71159	10.71763	2.37	0.018	1.507202	76.1265
SNPrs3914468_g	30.96926	41.30342	2.57	0.010	2.268251	422.8347
SNPrs4668135_a	133.5783	261.5591	2.50	0.012	2.877411	6201.117
SNPrs3815679_t	548.6712	1410.554	2.45	0.014	3.556284	84650.17
SNPrs58599996_a	125.8762	210.2276	2.90	0.004	4.768038	3323.131
SNPrs112277758_c	12.85109	13.90295	2.36	0.018	1.541941	107.1056
SNPrs140297819_g	125.8762	210.2276	2.90	0.004	4.768038	3323.131
SNPrs13026171_t	12.851	13.90278	2.36	0.018	1.541947	107.1037
SNPrs12692897_g	32.10611	47.16342	2.36	0.018	1.803784	571.4666
SNPrs12692900_g	32.10611	47.16342	2.36	0.018	1.803784	571.4666
SNPrs12622978_c	1844.766	5057.653	2.74	0.006	8.555938	397754.4

Women

SNPrs2024481_a	.1457287	.0930145	-3.02	0.003	.0417107	.5091465
SNPrs2229265_t	.2081133	.1227268	-2.66	0.008	.0655144	.661094
SNPrs56325975_c	.0782228	.0773183	-2.58	0.010	.0112713	.5428659
SNPrs13396247_a	.1027846	.0864311	-2.71	0.007	.0197768	.5341952
SNPrs9287914_a	.1486556	.1058031	-2.68	0.007	.0368428	.5998044
SNPrs3770616_t	.1038038	.0864259	-2.72	0.007	.0203008	.5307793
SNPrs7575260_a	.1038038	.0864259	-2.72	0.007	.0203008	.5307793
SNPrs4667600_a	.1839065	.1257039	-2.48	0.013	.0481707	.7021194
SNPrs3914468_g	.159003	.1116665	-2.62	0.009	.0401434	.6297917
SNPrs2892802_t	3.88091	2.219388	2.37	0.018	1.265186	11.90455
SNPrs700550_c	4.003154	1.974007	2.81	0.005	1.522873	10.52303
**SNPrs830966_g**	**4.281696**	**1.896411**	**3.28**	**0.001**	**1.797253**	**10.20052**
SNPrs830967_a	3.993804	1.968272	2.81	0.005	1.520155	10.49266
SNPrs830968_t	3.993804	1.968272	2.81	0.005	1.520155	10.49266
SNPrs830969_a	3.935537	1.938095	2.78	0.005	1.499067	10.33206
SNPrs831030_t	.3433465	.1565591	-2.34	0.019	.1404759	.8391962
SNPrs2673151_t	5.17998	3.171477	2.69	0.007	1.560178	17.19815
SNPrs830943_a	4.773296	2.569862	2.90	0.004	1.661674	13.71169
SNPrs2107301_a	3.856908	1.789459	2.91	0.004	1.55351	9.575567
SNPrs2239181_c	3.554051	1.78874	2.52	0.012	1.32531	9.530814
SNPrs2239180_g	3.554051	1.78874	2.52	0.012	1.32531	9.530814
SNPrs12721397_g	4.722825	2.473695	2.96	0.003	1.691859	13.18376
SNPrs2238135_g	2.709058	1.142603	2.36	0.018	1.185239	6.191997
**SNPrs4516035_c**	**.0003625**	**.0007642**	**-3.76**	**0.000**	**5.82e-06**	**.0225664**
**SNPrs7139166_g**	**.0003625**	**.0007642**	**-3.76**	**0.000**	**5.82e-06**	**.0225664**
**SNPrs10875696_t**	**.0003674**	**.0007739**	**-3.76**	**0.000**	**5.92e-06**	**.0228039**
**SNPrs11614332_a**	**.0003694**	**.000779**	**-3.75**	**0.000**	**5.92e-06**	**.0230531**
SNPrs11568820_c	.1061112	.080666	-2.95	0.003	.0239153	.470811
**SNPrs11168297_a**	**.0003625**	**.0007642**	**-3.76**	**0.000**	**5.82e-06**	**.0225664**
**SNPrs7310552_g**	**.0003625**	**.0007642**	**-3.76**	**0.000**	**5.82e-06**	**.0225664**
SNPrs7976091_c	.0598771	.0538288	-3.13	0.002	.0102811	.3487228

******************Baseline MetS************************

Overall:

SNPrs2268368_t |	.6272605	.1115087	-2.62	0.009	.4427199	.888724
SNPrs2268366_a |	.6272605	.1115087	-2.62	0.009	.4427199	.888724

Men:

SNPrs11674531_g |	2.07313	.6380819	2.37	0.018	1.134065	3.789789
SNPrs17848195_a |	.3748364	.1536393	-2.39	0.017	.1678604	.8370191
SNPrs2673163_c |	2.291486	.7618774	2.49	0.013	1.194285	4.396694
SNPrs2244407_t |	2.291486	.7618774	2.49	0.013	1.194285	4.396694
SNPrs2673166_c |	.4201989	.1413911	-2.58	0.010	.2172888	.8125918
SNPrs13002827_c |	3.755142	1.699488	2.92	0.003	1.546653	9.117168
SNPrs4566330_g |	2.051286	.5506439	2.68	0.007	1.21208	3.471534
SNPrs34155127_g |	3.813479	2.059483	2.48	0.013	1.323204	10.99046
SNPrs34996026_t |	2.837031	1.127943	2.62	0.009	1.301501	6.184203
SNPrs2239182_t |	.4611191	.1358182	-2.63	0.009	.2588809	.8213463
SNPrs7965266_t |	2.3329	.8448625	2.34	0.019	1.147182	4.744165
SNPrs7965274_t |	2.3329	.8448625	2.34	0.019	1.147182	4.744165
SNPrs7979131_g |	2.3329	.8448625	2.34	0.019	1.147182	4.744165
SNPrs2853566_g |	2.378857	.748435	2.75	0.006	1.283996	4.407304
SNPrs2853565_g |	2.700805	.8628076	3.11	0.002	1.443995	5.051504
SNPrs3922882_g |	2.512153	.9136143	2.53	0.011	1.231637	5.124001

Women:

SNPrs2268368_t |	.5632227	.1294154	-2.50	0.012	.3589996	.8836215
SNPrs2268366_a |	.5632227	.1294154	-2.50	0.012	.3589996	.8836215
SNPrs11902433_c |	1.813431	.434342	2.49	0.013	1.134035	2.899851
SNPrs34951037_a |	1.74467	.4068454	2.39	0.017	1.104635	2.755547
SNPrs3821127_a |	1.840489	.4462228	2.52	0.012	1.144356	2.960092
SNPrs6752778_a |	1.723875	.3904727	2.40	0.016	1.105863	2.687264
SNPrs2229267_a |	1.760211	.3855984	2.58	0.010	1.14577	2.704158
SNPrs1989969_a |	1.756767	.412795	2.40	0.016	1.108418	2.784355

***********Follow-up MetS****************

**********Overall***********

SNPrs11574044_c |	.4781762	.1113382	-3.17	0.002	.3029666	.7547117
SNPrs4760603_t |	2.002399	.5215423	2.67	0.008	1.201843	3.336211

**********Men****************

SNPrs146901930_a |	.1370364	.1142064	-2.38	0.017	.0267573	.7018253
SNPrs17848195_a |	.3190816	.1515506	-2.41	0.016	.1257813	.8094452
SNPrs73971315_g |	.3646399	.1478522	-2.49	0.013	.1647114	.8072439
SNPrs2673175_t |	.2401759	.1395053	-2.46	0.014	.0769329	.7498022
SNPrs2544386_a |	.2211334	.1100727	-3.03	0.002	.0833595	.5866158
SNPrs141248390_a |	.2220275	.1105593	-3.02	0.003	.0836658	.5892035

SNPrs830994_g |	.3883239	.1400447	-2.62	0.009	.1915216	.7873549
SNPrs830995_a |	.3682697	.1397929	-2.63	0.008	.1750069	.7749555
SNPrs830998_a |	.3009102	.1427092	-2.53	0.011	.1187811	.7623009
SNPrs831000_t |	.3009102	.1427092	-2.53	0.011	.1187811	.7623009
SNPrs831001_t |	.3009102	.1427092	-2.53	0.011	.1187811	.7623009
SNPrs831002_t |	.2931144	.1386247	-2.59	0.009	.1160038	.7406312
SNPrs2544372_c |	.2983195	.1410801	-2.56	0.011	.1180687	.7537521
SNPrs831027_t |	3.567771	1.587104	2.86	0.004	1.491916	8.531971
SNPrs78991977_g |	4.066832	1.884048	3.03	0.002	1.640283	10.08309
SNPrs16856823_t |	2.920439	1.263456	2.48	0.013	1.250825	6.818669
SNPrs3770636_g |	2.920439	1.263456	2.48	0.013	1.250825	6.818669
SNPrs4566330_g |	2.145472	.6391648	2.56	0.010	1.196571	3.846867
SNPrs34155127_g |	5.893035	3.532662	2.96	0.003	1.820012	19.08111
SNPrs4760648_c |	2.492837	.8844024	2.57	0.010	1.243675	4.996674
SNPrs2853566_g |	2.292185	.796091	2.39	0.017	1.160437	4.527699

********<u>**Women**</u>************

SNPrs2673165_t |	1.757246	.3646304	2.72	0.007	1.170057	2.639111
SNPrs13396247_a |	.5030988	.1317353	-2.62	0.009	.3011397	.8405014
SNPrs2673164_t |	1.728496	.3595509	2.63	0.009	1.149759	2.598544
SNPrs9287914_a |	.5643848	.1370892	-2.35	0.019	.3506052	.9085155
SNPrs2544373_t |	1.630716	.3299878	2.42	0.016	1.09681	2.424518
SNPrs700550_c |	2.024985	.5177484	2.76	0.006	1.226836	3.34239
SNPrs830966_g |	1.951917	.4499172	2.90	0.004	1.242394	3.066645
SNPrs830967_a |	2.01782	.5160316	2.75	0.006	1.222358	3.330936
SNPrs830968_t |	2.01782	.5160316	2.75	0.006	1.222358	3.330936
SNPrs830969_a |	1.902251	.4857009	2.52	0.012	1.15327	3.137652
SNPrs853988_c |	2.031602	.5290717	2.72	0.006	1.219461	3.384616
SNPrs830972_g |	2.031602	.5290717	2.72	0.006	1.219461	3.384616
SNPrs11574114_t |	.4187679	.151039	-2.41	0.016	.2065221	.8491417
SNPrs2107301_a |	2.032922	.5675101	2.54	0.011	1.17625	3.513517

Note: Criterion for reporting results: p<0.01 (overall) and p<0.02 per gender group: Criterion for noteworthy results> p≤0.001 (bolded).
